# An angular motion of a conserved four-helix bundle facilitates alternating access transport in the TtNapA and EcNhaA transporters

**DOI:** 10.1073/pnas.2002710117

**Published:** 2020-11-30

**Authors:** Gal Masrati, Ramakanta Mondal, Abraham Rimon, Amit Kessel, Etana Padan, Erik Lindahl, Nir Ben-Tal

**Affiliations:** ^a^Department of Biochemistry and Molecular Biology, George S. Wise Faculty of Life Sciences, Tel Aviv University, Ramat Aviv 69978, Israel;; ^b^Department of Biological Chemistry, The Alexander Silberman Institute of Life Sciences, The Hebrew University of Jerusalem, 91904 Jerusalem, Israel;; ^c^Science for Life Laboratory, Stockholm University & KTH Royal Institute of Technology, 171 65 Solna, Sweden

**Keywords:** cation/proton antiporters, rocking bundle mechanism, elevator mechanism, molecular dynamics, NhaA

## Abstract

There is ongoing debate regarding the mechanism through which cation/proton antiporters (CPAs), like *Thermus thermophilus* NapA (TtNapA) and Escherichia coli NapA (EcNhaA), alternate between their outward- and inward-facing conformations in the membrane. CPAs comprise two domains, and it is unclear whether the transition is driven by their rocking-bundle or elevator motion with respect to each other. Here we address this question using metadynamics simulations of TtNapA, where we bias conformational sampling along two axes characterizing the two proposed mechanisms: angular and translational motions, respectively. By applying the bias potential for the two axes simultaneously, as well as to the angular, but not the translational, axis alone, we manage to reproduce each of the two known states of TtNapA when starting from the opposite state, in support of the rocking-bundle mechanism as the driver of conformational change. Next, starting from the inward-facing conformation of EcNhaA, we sample what could be its long-sought-after outward-facing conformation and verify it using cross-linking experiments.

## Significance

Membrane-embedded cation/proton antiporters (CPAs) exchange monovalent cations with protons by alternating between two extreme states: inward- and outward-facing. Although atomic-resolution structures are available, there is still an ongoing debate regarding these antiporters’ transport mechanism. Here, we use computer simulations to explore the dynamics of two bacterial antiporters along two axes of motion: a tilting movement and a vertical translation of the antiporters’ two functional domains. By analyzing these dynamic changes, rather than comparing the two extreme states that CPAs adopt, we determine that it is the tilting movement of the antiporters’ mobile domain that drives the relevant conformational changes. Finally, by applying the knowledge gained from these simulations, we predict an unknown outward-facing conformation and verify it experimentally.

A secondary active transporter harnesses the electrochemical gradient created by the movement of an ion across a biological membrane to facilitate the transport of another ion or molecule against its electrochemical gradient. Structural data obtained throughout the past decade have revealed that these transporters share some common structural features and can be classified under three main structural folds: the MFS fold (major facilitator superfamily), the LeuT fold (leucine transporters), and the NhaA fold (Na^+^/H^+^ antiporters) (1, 2).

When folded, these transporters are organized in two functional domains: 1) a mobile core domain that harbors an ion-binding site and 2) a dimerization/oligomerization domain (1). To carry out their function, they alternate between at least two main states—inward-facing and outward-facing (IF and OF) —such that their binding sites are accessible from different sides of the membrane in each state in what is known as an alternating access model (3). Extensive research has been devoted to elucidating the mechanisms underlying the alternating access model in secondary active transporters. In particular, studies of the LeuT fold have suggested that a four-helix bundle in the core domain undergoes significant conformational changes during transport (4). Notably, this four-helix bundle is structurally conserved across many transporters, suggesting that it may be implicated in the conformational changes underlying their function as well.

**Fig. 1.**
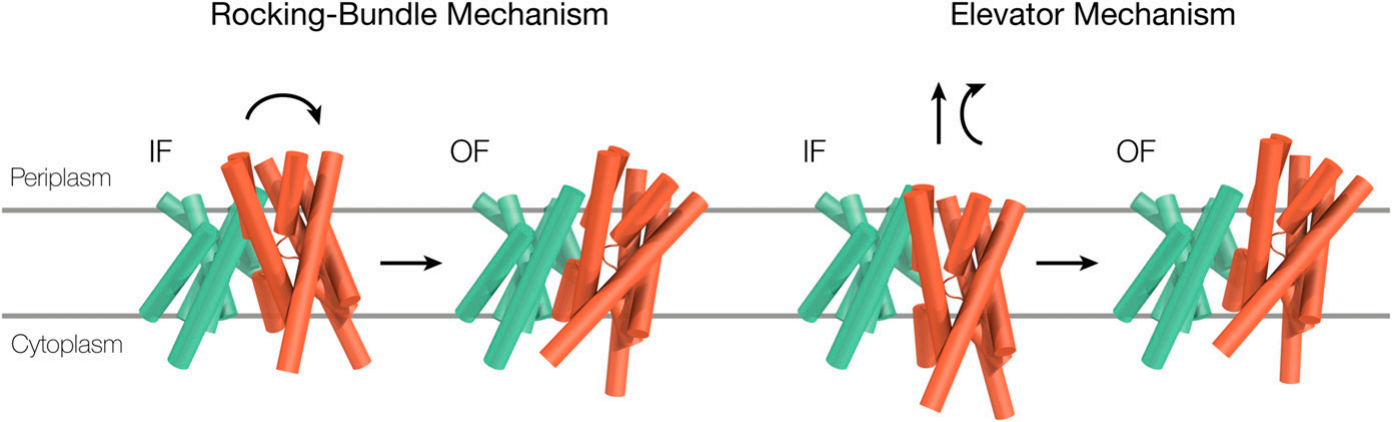
Rocking bundle vs. elevator mechanism. Schematic representation of TtNapA oriented in the membrane such that the periplasm is at the top and the cytoplasm is at the bottom. Transmembrane helices are shown as cylinders, where the core domain is designated in orange and the dimerization domain in green. The core domain’s direction of movement relative to the dimerization domain that correlates with each of the two mechanisms underlying the alternating access model for cation/proton transport is marked with black arrows.

Herein, using simulations, we contribute new insights with regard to the alternating access mechanism of the cation/proton antiporter (CPA) superfamily of secondary active transporters. CPAs exchange monovalent cations with protons, and their malfunction is associated with a growing number of human pathologies (5) affecting millions worldwide. However, there is still an ongoing debate regarding the structural mechanism underlying the alternating access model in CPAs (6, 7). One possibility is a rocking-bundle mechanism, which involves a tilting movement of the core domain relative to a stationary dimerization domain on the plane of the membrane; this mechanism was originally proposed on the basis of the structure of the bacterial LeuT transporter (4) (Fig. 1). An alternative possibility is the elevator mechanism, first described in a bacterial homolog of the glutamate transporter Glt_Ph_. Glt_Ph_ is a member of the proton/sodium glutamate symport protein fold (1) that shares some similar structural features with the MFS, LeuT, and NhaA folds. This mechanism involves a prominent vertical movement of the transporter’s core domain along the membrane’s normal, which is accompanied by a tilting movement (8) (Fig. 1).

Thus far, three-dimensional structures of four CPAs have been determined experimentally (6, 9–11). Most notably, the structure of the CPA member NapA from *Thermus thermophilus* (TtNapA) was solved in both IF and OF states (6). Consistent with observations obtained for LeuT (4), these structures revealed that the main rearrangements between the alternating states of TtNapA occur in the conserved four-helix bundle in the core domain (6). This bundle includes the two discontinuous transmembrane helices (TMs) 4 and 11 (2), as well as TM-5, which includes the ion-binding site, and TM-12; the numbering of the helices follows the prevalent helix nomenclature of *Escherichia coli* NhaA (EcNhaA), which will be used throughout the text (12). A comparison of the two oppositely facing structures of TtNapA reveals an angular motion of one domain relative to the other that is also accompanied by a 6-Å vertical displacement of the antiporter’s core domain relative to the membrane and 7 Å relative to the dimer domain (6). This is consistent with the elevator mechanism proposed for Glt_Ph_ that involves the two types of motions. It was thus suggested that the structural data favor the elevator mechanism in CPAs.

While this interpretation is straightforward, the extreme conformations represented by the crystal structures do not provide any direct information about the actual conformational changes that take place during the transport processes. Accordingly, on its own, the structural information obtained for TtNapA is insufficient for determining whether the angular motion, vertical translation, or both underlie the alternating access model of cation/proton antiport in CPAs. Okazaki et al. (13) used molecular-dynamics (MD) simulations to predict the transition path between the IF and OF conformations of an archaeal member of the CPA superfamily. Their work demonstrated how insightful simulations can be in CPAs, but it did not address the question of which mode of motion is essential for the IF–OF transition.

Here, we used metadynamics, an enhanced sampling MD protocol, to study the conformational dynamics of two CPA members, TtNapA and EcNhaA, embedded in a lipid bilayer. We focused on the structurally conserved four-helix bundle in an attempt to predict which mode of motion is most likely to drive the IF–OF transition in CPAs. Specifically, we applied external potential to bias the conformational sampling along the two axes of motion in TtNapA that underlie the rocking-bundle and elevator mechanisms. The first axis corresponds to an angular motion of the conserved four-helix bundle in the antiporter’s core domain relative to a separate four-helix bundle in the dimerization domain, and the second axis corresponds to a vertical translation of the four-helix bundle along the membrane normal. By applying the bias potential for both axes simultaneously, as well as for the angular motion axis alone, we successfully reproduced each of the two known states of TtNapA when starting from the opposite state, with accuracy of between 0.80 and 1.51 Å. Importantly, although both types of motions are observed, our results suggest that conformational changes depend mainly on the angular motion rather than on the translation. This dominance of the angular motions in determining the IF-to-OF transition is more in line with the rocking-bundle mechanism of ion/proton antiport. Furthermore, by biasing the conformational sampling of the IF conformation of EcNhaA along the angular motion axis, we sampled a conformation that seems to fit the long-sought-after OF conformation of this protein, and we verified it experimentally. Accordingly, in addition to providing a better understanding of the transport mechanism of CPAs, our findings point to the potential of computational methods to predict, for example, additional conformations for CPA members with only one known experimental structure, as well as to obtain more metastable states.

## Results

### Metadynamics Simulations.

To study the mechanism underlying the alternating access model in TtNapA [Protein Data Bank (PDB) entries 5BZ2 and 5BZ3] (6) and in EcNhaA (PDB entry 4AU5) (9), we employed well-tempered metadynamics simulations (14) of the proteins embedded in a mixed phosphatidylethanolamine and phosphatidylglycerol lipid bilayer.

**Fig. 2.**
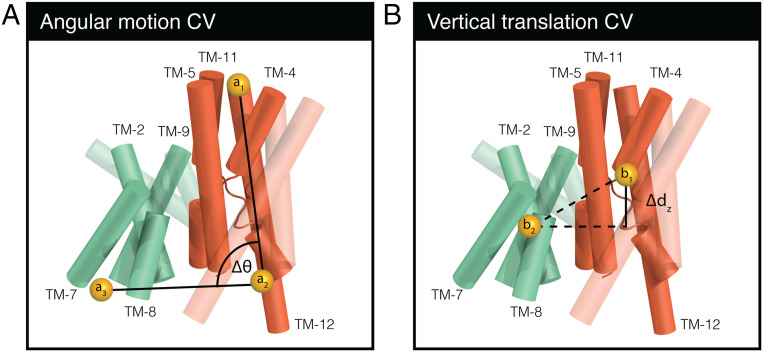
Angular-motion and vertical-translation CVs. Schematic representation of TtNapA’s transmembrane helices as cylinders, with TM-6 and the loops removed for clarity. The core domain is colored orange and the dimerization domain green, with the four-helix bundles in each domain highlighted and designated by their respective helix numbers. (*A*) The angular-motion CV, represented as the change in angle (Δθ) between the conserved four-helix bundle in the antiporter’s core domain (highlighted in orange) and a four-helix bundle in the dimerization domain (highlighted in green). θ is defined by three virtual atoms: a_1_ and a_2_ at the periplasmic and cytoplasmic ends of the four-helix bundle and a_3_ at the cytoplasmic end of the four-helix bundle of the dimerization domain. (*B*) The vertical-translation CV represented by the change in distance between the two four-helix bundles along the membrane normal (Δd_z_). Δd_z_ is defined by two virtual atoms (b_1_ and b_2_) representing the center of mass of each bundle. A more detailed description of the two CVs is provided in the *SI Appendix*.

Metadynamics simulations are based on the assumption that a process can be approximated by a few degrees of freedom, known as collective variables (CVs). By applying history-dependent external bias potential along a given set of CVs, metadynamics discourages the return of the system to states that were already sampled. This approach thus enhances the conformational sampling along the chosen CVs. As the amount of bias introduced into the system is known, metadynamics also allows for computing the free energy landscape as a function of the CVs (14). Here, we applied the external bias potential to two CVs describing the two modes of motion that underlie the rocking-bundle and elevator mechanisms. Specifically, we biased the change in angle of the conserved four-helix bundle and its vertical translation along the membrane normal (Fig. 2). In total, 19 independent simulations of 1 to 1.5 µs each were conducted for TtNapA, along with four 0.6-µs simulations for EcNhaA, starting from either the IF or OF state (*SI Appendix*, Table S1). A more detailed description is provided in *Materials and Methods* and the *SI Appendix*.

### Two-CV Metadynamics Simulations of TtNapA.

As elaborated in the Introduction, data obtained from the crystal structures of the IF and OF conformations of TtNapA suggest that the shift between the two states entails both an angular motion and a vertical translation of the core domain relative to the dimerization domain, where the most prominent differences between the two conformations are observed in the core domain’s four-helix bundle (6). Thus, in an attempt to reproduce an accurate sampling of the conformational space, we simultaneously biased the CVs corresponding to the angular motion and to the vertical translation. In four independent simulations—two starting from the IF conformation of TtNapA and two from the OF conformation—biasing both CVs enabled the antiporter to alternate between states.

To estimate the free energy associated with the above conformational changes, we used the HISTOGRAM function in PLUMED (15). In this procedure, the data collected during the four simulations were reweighted, and the probability density function of each CV was computed (16). The free energy was then estimated using the Boltzmann distribution:

$$F\left(x\right)=-k_{B}T\ln\left(H\left(x\right)\right),$$ [1]

where *F*(*x*) is the free energy, *k*_*B*_*T* is the product of the Boltzmann constant and the temperature, and *H*(*x*) is the histogram representing the probability density function. Finally, the average error was estimated from block analysis with four equal blocks, one for each simulation.

**Fig. 3.**
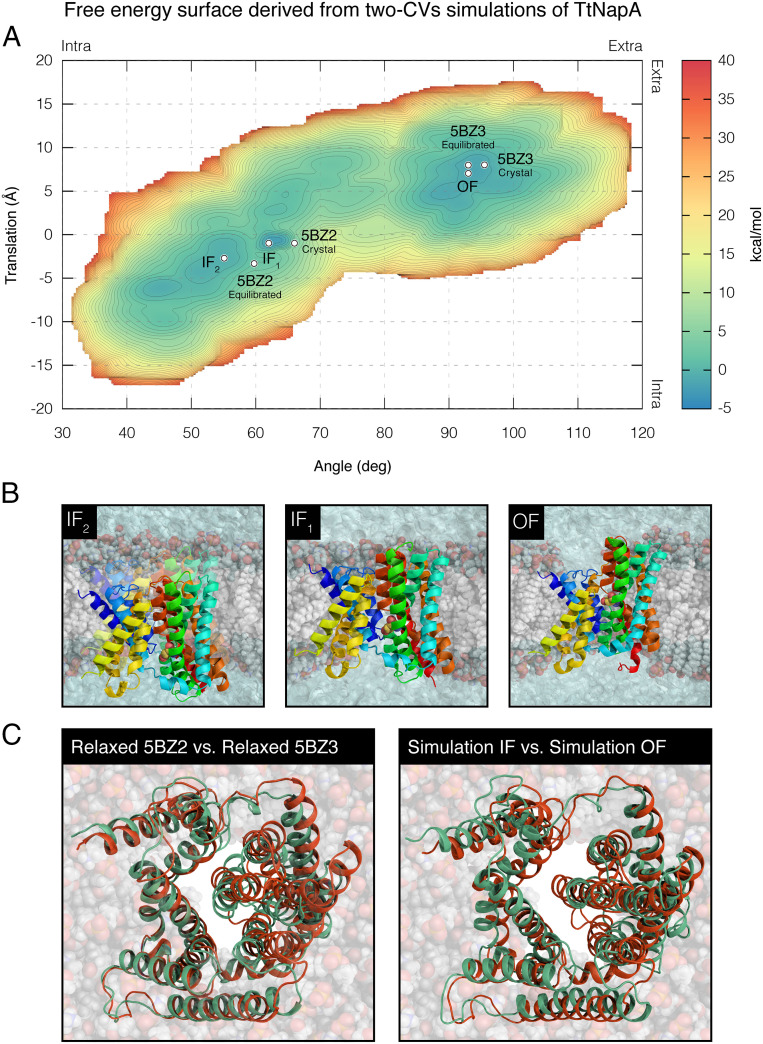
Free energy surface derived from two-CV simulations of TtNapA. (*A*) The estimated free energy landscape associated with the conformational sampling of TtNapA as a function of vertical translation (*y* axis) and angular motion (*x* axis). Blue-to-orange color gradient represents low to high free energy, respectively, with an average error of 0.2 kcal/mol. The three main free energy minima, IF_1_, IF_2_, and OF, and the location of TtNapA’s crystal structures and equilibrated crystal structures are marked with white circles. The direction of movement with respect to the extracellular and intracellular sides of the membrane is indicated at the top and right edges of the free energy landscape. (*B*) Vertical dissections of the lipid bilayer exposing the antiporter in three different conformations corresponding to the three free energy minima. TM-6 and lipids that correlated with the IF or OF funnels were removed to visualize the accessibility of D157, shown in sphere representation, known to interact with the substrate ion. (*C*) Superimposition of TtNhaA’s equilibrated IF (PDB ID code 5BZ2; colored green) and OF (PDB ID code 5BZ3; colored orange) states in cartoon representation as seen from the periplasm (*Left*) compared to the superimposition of the simulation-derived IF and OF states (*Right*; green and orange, respectively).

The resulting free energy landscape revealed two distinct basins, corresponding to the IF and OF states, separated by a barrier, which, at its lowest point, is 9.8 kcal/mol (average error of ±0.2 kcal/mol; Fig. 3*A*). The IF basin appears to be slightly wider than the OF basin, potentially containing two local minima, denoted as IF_1_ and IF_2_. IF_1_ presents a slightly shallower free energy surface compared to IF_2_ (Fig. 3*A*). In IF_2_, the four-helix bundle in the core domain is more tilted and translated compared with that in IF_1_, such that, in IF_2_, the binding site is somewhat more exposed to the cytoplasm (Fig. 3*B*). A comparison between the IF and OF conformations in the simulations indicates that the main structural rearrangements occur in the four-helix bundle, with the two remaining helices of the core domain (TM-3 and TM-10) moving mainly along the angular motion axis in the plane of the membrane (Fig. 3*C*). This is consistent with observations from the crystal structures of the transporter in the two oppositely facing conformations. Importantly, the simulations managed to reproduce each of the two crystal structures of TtNapA deposited in the PDB when starting from the opposite state, with backbone rmsd values of 1.12 Å for the IF state (PDB entry 5BZ2) and 1.39 Å for the OF state (PDB entry 5BZ3), excluding loop regions due to their inherent conformational plasticity (*SI Appendix*, Fig. S1 *A* and *B*). The equilibrated crystal structures were reproduced with backbone rmsd values of 1.05 Å for the IF state and 1.07 Å for the OF state (*SI Appendix*, Fig. S1 *C* and *D*).

### Single-CV Metadynamics Simulations of TtNapA.

To determine the individual contributions of each CV, we carried out metadynamics simulations in which only one CV was biased and the system’s conformational sampling was monitored. As shown in *SI Appendix*, Fig. S2, biasing the angular-motion CV alone enabled the antiporter to alternate between the IF and OF states (Movie S1). In contrast, biasing the vertical-translation CV enabled the system to alternate between states in only one out of four simulations (*SI Appendix*, Fig. S3). Importantly, despite a massive translation of up to 18 Å of the core domain’s four-helix bundle relative to the initial state of the simulation, transition between states only occurred when the translation was accompanied by an angular motion, which happened spontaneously in one of the simulations (*SI Appendix*, Fig. S3 and Table S1). For the latter simulation, we examined the vertical-translation CV before and after the transition point and observed little difference (*SI Appendix*, Fig. S3*F*, time frames 0.35 to 0.45 µs), suggesting that the conformational changes were independent of the vertical movement along the membrane’s normal.

**Fig. 4.**
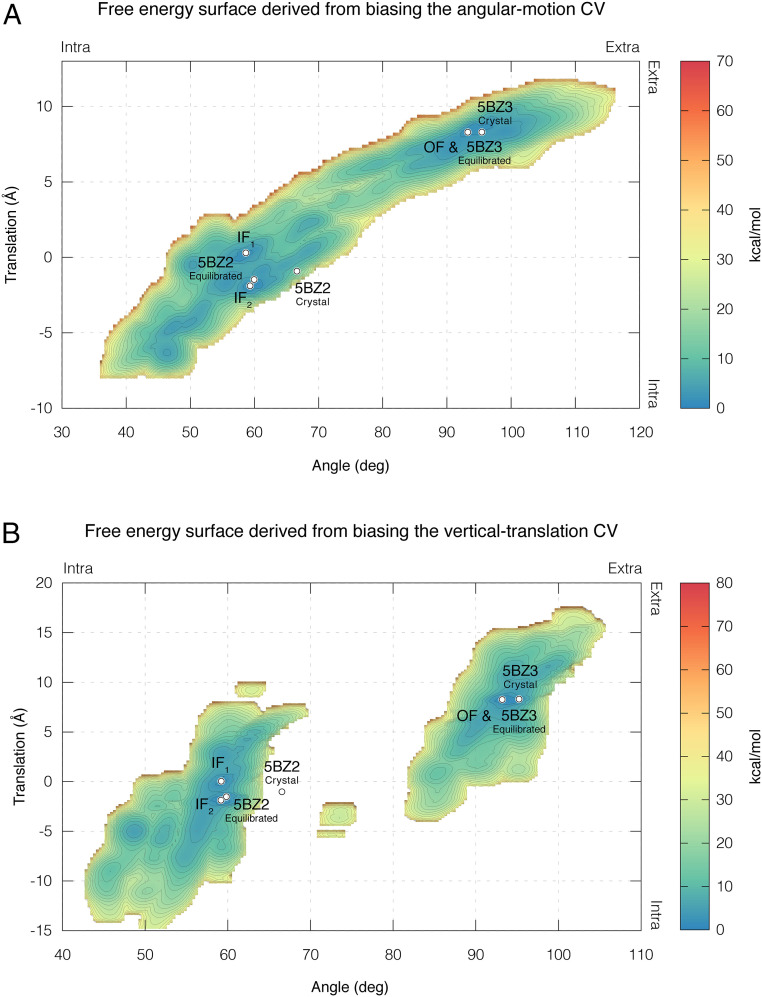
Free energy surface derived from single-CV simulations of TtNapA. The estimated free energy landscape associated with the conformational sampling of TtNapA as a function of vertical translation (*y* axis) and angular motion (*x* axis) derived from simulations where only the angular-motion CV was biased (*A*) or simulations where only the vertical-translation CV was biased (*B*). Blue-to-orange color gradient represents low to high free energy, respectively, with an average error of 0.2 kcal/mol. The three main free energy minima, IF_1_, IF_2_, and OF, and the location of TtNapA’s crystal structures and equilibrated crystal structures are marked with white circles. Notably, the free energy landscape derived from the angular-motion CV simulations (*A*) is comparable to that derived from the two-CV simulations (Fig. 3). Importantly, in terms of the angular motion and vertical translation, the same free energy minima and same barrier were retrieved.

As both the angular-motion and the vertical-translation CVs were monitored during the single-CV simulations, we used the collected data to compute the free energy landscape as a function of both CVs. The resulting free energy landscapes show that, in contrast to the case of the vertical-translation CV, biasing the angular-motion CV alone is sufficient to reproduce a free energy landscape that is similar to the one obtained when biasing both CVs simultaneously (Fig. 4). Notably, in terms of the angular motion and vertical translation values, the three main free energy minima (IF_1_, IF_2_, and OF) and the free energy barrier are highly similar (Figs. 3 and 4). In terms of the energy, these simulations produced a higher free energy barrier of at least 16 kcal/mol (average error of ±0.2 kcal/mol). Once more, the simulations, although untargeted, managed to reproduce each of the two crystal structures of the antiporter deposited in the PDB when starting from the opposite state, with backbone rmsd values of 1.51 Å for the IF conformation and 1.17 Å for the OF conformation (*SI Appendix*, Fig. S4 *A* and *B*). The equilibrated crystal structures were reproduced with backbone rmsd values of 1.2 Å for the IF state and 0.8 Å for the OF state (*SI Appendix*, Fig. S4 *C* and *D*).

### EcNhaA.

The above results suggest that biasing the angular motion of the core domain’s four-helix bundle is sufficient to induce the relevant conformational changes in TtNapA. Thus, we applied the same approach to the IF crystal structure of EcNhaA (PDB entry 4AU5) in order to produce a model structure of its OF conformation, which is currently unknown. Specifically, we biased the angular-motion CV starting from the IF crystal structure and monitored the conformational sampling. Here too, the angular motions allowed the antiporter to alternate between states, and it adopted an OF conformation, which arguably suggests the transporters have evolved to facilitate a relatively simple conformational transition. To improve sampling, we enabled one of the simulated OF conformations to relax for 150 ns in an unbiased simulation. Then, we conducted another metadynamics simulation of EcNhaA starting from the OF equilibrated model to verify that the procedure can reproduce the IF conformation when used in the opposite direction.

**Fig. 5.**
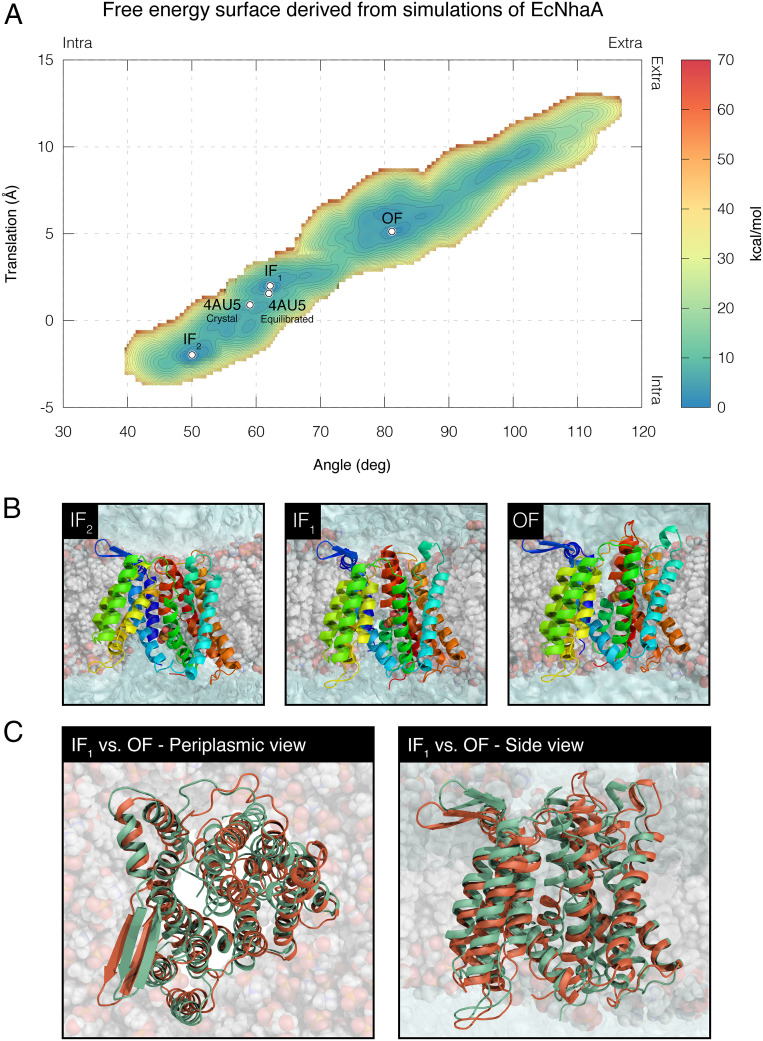
Free energy surface derived from simulations of EcNhaA. (*A*) The estimated free energy landscape associated with the conformational sampling of EcNhaA as a function of vertical translation (*y* axis) and angular motion (*x* axis). The free energy was derived from single-CV simulations, where only the angular-motion CV was biased. Blue-to-orange color gradient represents low to high free energy, respectively, with an average error of ±0.2 kcal/mol. The three main free energy minima, IF_1_, IF_2_, and OF, and the location of EcNhaA’s crystal structure and equilibrated crystal structure are marked with white circles. The direction of movement with respect to the extracellular and intracellular sides of the membrane is indicated at the top and right edges of the free energy surface. (*B*) Vertical dissections of the lipid bilayer exposing the antiporter in three different conformations corresponding to the three free energy minima. TM-6 and lipids that correlated with the IF or OF funnels were removed to visualize the water accessibility of D164, shown in sphere representation. The latter is known to interact with the substrate ion. (*C*) Superimposition of EcNhaA’s IF_1_ and OF states in cartoon representation as seen from the periplasm (*Left*) and in the plane of membrane (*Right*). The IF state is colored green and the OF state orange.

The results, presented in Fig. 5, reveal a free energy landscape similar to that obtained for TtNapA. Interestingly, the landscape features three free energy basins, representing two IF states (IF_1_ and IF_2_) and one OF state. The IF states are separated from the OF state by an energy barrier of at least 11.4 kcal/mol (average error of ±0.2 kcal/mol). Starting from the OF model, the simulation managed to reproduce the IF crystal structure of EcNhaA (PDB entry 4AU5) with a backbone rmsd of 1.36 Å, excluding loop regions (*SI Appendix*, Fig. S5*A*). The equilibrated crystal structure was reproduced with a backbone rmsd of 1.55 Å (*SI Appendix*, Fig. S5*B*). Theoretically, it is possible that the OF model still “has memory” of the original IF conformation. While this is possible for EcNhaA, it is certainly not the case for TtNapA, where simulations initiated from the crystal structure of the OF conformation successfully reproduced that of the IF conformation (and vice versa). We believe that the same is true for EcNhaA.

### Model-Based Cysteine Cross-Linking in EcNhaA.

To determine the validity of the proposed conformation, we designed an NhaA variant with two Cys replacements at positions G15 (TM-1) and A142 (TM-4). These positions, in sufficient spatial proximity only in the suggested OF conformation (*SI Appendix*, Fig. S6 *A* and *B*), and the formation of a cross-link between them under oxidizing conditions, would support this conformation. This can easily be measured, as the formation of the cross-link would lock the antiporter in an inactive state, and activity should be restored under reductive conditions. To ensure that the protein is in its monomeric state, as in the simulations, we introduced the cysteine replacements to a Cys-less NhaA variant lacking a β-sheet between TM-1 and TM-2 that is crucial for dimerization [Δ(P45-N58)-CL-NhaA]. This mutant has been previously shown to be a fully functional NhaA monomer (17).

Three mutants were constructed using Δ(P45-N58)-CL-NhaA in a pCL-AXH3 plasmid, including two single mutants, Δ(P45-N58)-G15C-CL-NhaA and Δ(P45-N58)-A142C-CL-NhaA, and one double mutant, Δ(P45-N58)-G15C-A142C-CL-NhaA. For simplicity, these are denoted as G15C, A142C, and G15C-A142C, respectively.

**Fig. 6.**
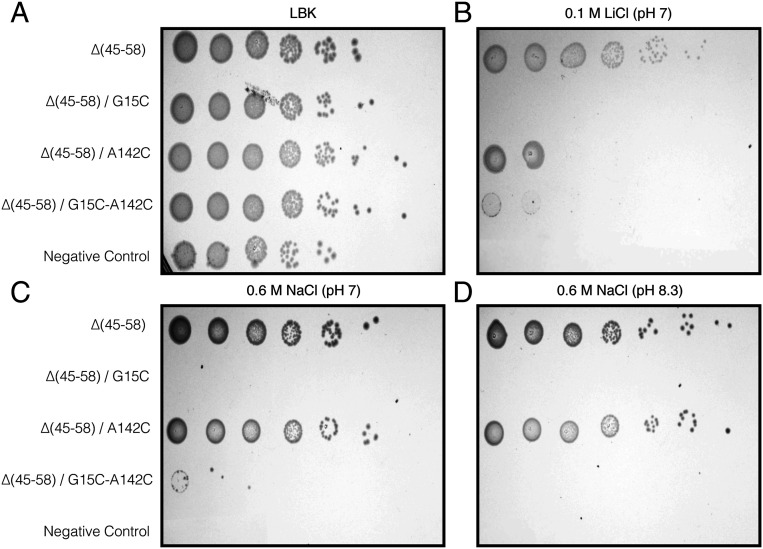
Growth phenotype of NhaA mutants. EP432 cells transformed with the monomeric CL-NhaA variant, ∆(45-58) or each of the following mutated monomeric NhaA: ∆(45-58)/G15C, ∆(45-58)/A142C, and ∆(45-58)/G15C-A142C, were grown under varying conditions of salt concentration and pH as indicated. Cells transformed with pBR322 plasmid were used as a negative control. (*A*) Cells grown on the nonselective medium [modified LB (LBK) in which NaCl was replaced with KCl] (18). (*B*) Cells’ resistance to 0.1 M Li^+^ under neutral pH. (*C* and *D*) Cells’ resistance to 0.6 M Na^+^ under neutral or alkaline (8.3) pH.

To determine the growth phenotypes of the mutants, the respective plasmids were transformed into EP432, an *E. coli* strain lacking the Na^+^/H^+^ antiporter genes *nha*A and *nha*B. This strain can grow on nonselective agar plates (LBK, a modified LB in which NaCl was replaced with KCl), but cannot grow on high-Na^+^/Li^+^ selective media (0.6 M NaCl at pH 7 or pH 8.3 and 0.1 M LiCl at pH 7) unless transformed with a plasmid bearing WT NhaA [Δ(P45-N58)-CL-NhaA; positive control]. EP432 cells transformed with pBR322 served as a negative control. The expression of the mutants in EP432 was similar to the WT. Δ(P45-N58) and A142C grew on all selective media, although the latter showed slightly impaired growth with Li^+^. Contrarily, the G15C and G15C-A142C mutants could only grow on the nonselective medium (LBK; Fig. 6).

**Fig. 7.**
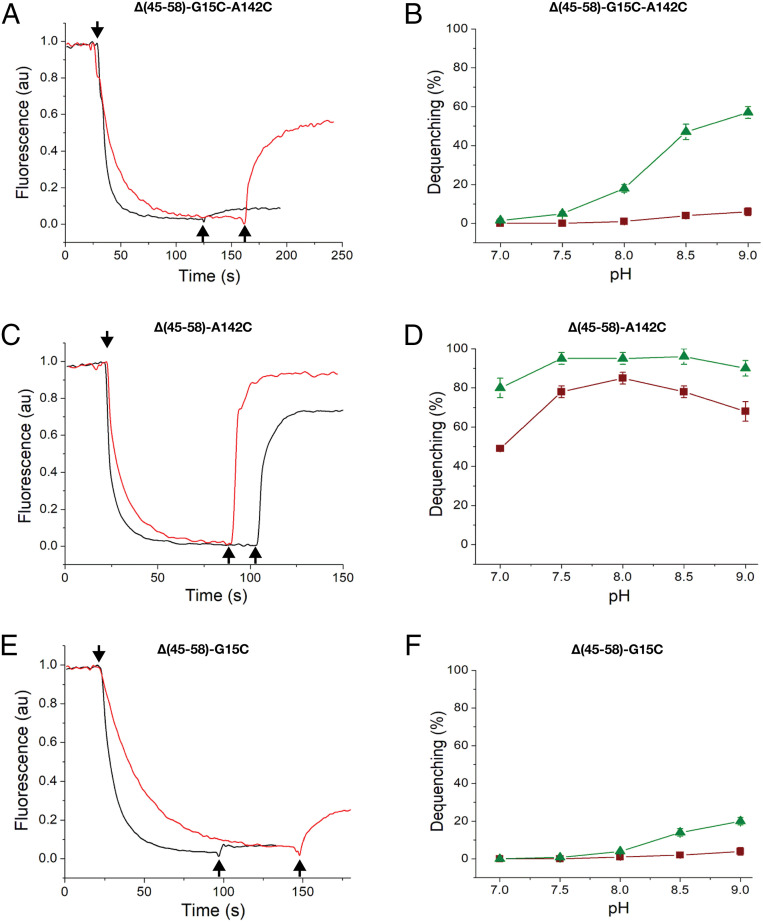
Na^+^/H^+^ antiporter activity in isolated membrane vesicles of NhaA mutants. Everted membrane vesicles were prepared from EP432 cells expressing the indicated NhaA variants grown in LBK (pH 7). Before the experiment, the reaction mixture was preincubated for 20 min in the presence (red) or absence (black) of 10 mM βME at pH 9 (*A*, *C*, and *E*) or at various pH values also in the presence (green) or absence (maroon) of 10 mM βME (*B*, *D*, and *F*). Na^+^/H^+^ antiporter activity was determined using acridine orange fluorescence to monitor ΔpH in the presence of Na^+^/Li^+^ (10 mM). The fluorescence traces [in arbitrary units (au)] are shown in the left column. After energization with D-lactate (2 mM, downward-pointing arrow), fluorescence was quenched and achieved a steady state. Then, 10 mM cation was added (upward-pointing arrow). A reversal of the fluorescence level (quenching) indicates that protons are exiting the vesicles in antiport with Na^+^/Li^+^. In the right column, the results are expressed in percentage of fluorescence dequenching following the addition of the cations. The SDs are shown in bars.

Next, we tested the mutants for transport activity in everted membrane vesicles under various pH values and under oxidizing or reductive conditions. As shown in Fig. 7 *A* and *B*, under oxidizing conditions, the double mutant (G15C-A142C) showed hardly any antiporter activity. However, addition of β-mercaptoethanol (βME) to the reaction mixture increased the antiporter activity dramatically (12 fold; Fig. 7*A*). Furthermore, the G15C-A142C double mutant showed a pH dependence similar to that of the WT (Fig. 7*B*). The antiporter is inactive below pH 7 and reaches maximum activity between pH 8 and 9 (19, 20).

We also measured the transport activity of each of the two single mutants (G15C and A142C). As the single mutants cannot cross-link intramolecularly, we expected their antiport activity to be unaffected by the oxidizing/reducing conditions. Indeed, the A142C mutant was virtually fully active with or without βME (Fig. 7 *C* and *D*). The G15C mutant was nearly inactive, as expected from its incapability to render the cells’ Na^+^/Li^+^/pH growth-resistant (Fig. 6). Addition of βME restored only a small portion of antiporter activity (Fig. 7 *E* and *F*). At this point, we cannot explain the small effect of βME on G15C, but, given the high evolutionary conservation of this position (*SI Appendix*, Fig. S6*C*), it may have other biological roles that explain the measurements.

## Discussion

Deciphering the conformational dynamics of CPAs is a necessary step toward obtaining a complete understanding of their transport mechanisms. Here, we used the accelerated MD protocol of metadynamics to test two mechanisms that have been proposed to underlie the alternating access model of cation/proton antiport in the CPA members TtNapA and EcNhaA. An external force was applied to bias the conformational sampling along coordinates (CVs), capturing the angular motion and vertical translation of the conserved four-helix bundle in each antiporter’s core domain relative to a corresponding bundle in the dimerization domain (Fig. 2). These two modes of motion underlie the two proposed transport mechanisms, i.e., the rocking-bundle and elevator mechanisms. Our results suggest that conformational changes depend mostly on the angular motion.

### Limitations.

It is important to note that the simulations were carried out in the absence of the bound transported ion/protons (21). However, the ion/protons–transporter interaction should only shift the balance between preexisting conformational equilibrium (22, 23), such that we would expect to observe similar conformations in the presence of an ion/protons. Reassuringly, simulations with proton-bound (four 1-µs simulations) and -unbound (four 1-µs simulations) transporters yielded energy landscapes with the same topology (*SI Appendix*, Fig. S7). Moreover, the estimated free energy surface obtained using the protonated vs. deprotonated forms were very similar. The very minor differences that were observed between the free energy surfaces, estimated from these two independent simulation datasets, further attests to the robustness of the approach, and the chosen CVs in particular.

In addition, our simulations simplify the inherently complex dynamics of conformational shifts in transporters by reducing the changes in multiple degrees of freedom to changes in only one or two. Specifically, the free energy values were calculated by sampling only the angular-motion and/or the vertical-translation CVs in order to reduce the computational cost. The molecule is still free to explore these other degrees of freedom, but they will be orthogonal to the CV sampling and averaged out in the analysis. It follows that this analysis is a qualitative approximation of the conformational sampling associated with the IF–OF transition in both antiporters. Indeed, the calculated height of the free energy barrier between the IF and OF states, ∼10 to ∼16 kcal/mol, might slightly underestimate experimentally derived values, which range between 14 and 18 kcal/mol (21, 24). It is noteworthy that our aim was to determine the mode of motion underlying the conformational changes in TtNapA and EcNhaA, rather than to accurately estimate the heights of the respective energy barriers. Nevertheless, despite all simplifications, our simulations succeed in capturing the structural changes that occur in both antiporters. This is reflected in the ability of the simulations to reproduce experimental structures with excellent accuracy. Such results are particularly impressive considering that the simulations started from the opposite state and were carried out in an untargeted manner, which adds further credibility to the applied approach, including the CVs selected. Based on these achievements, we made two important predictions. First, we predicted which of the two ion-transport mechanisms previously suggested for these transporters is most likely to be true; and, second, we predicted what could be the OF conformation of EcNhaA and provided experimental proof that this structure is viable.

### TtNapA.

As discussed in the Introduction, a comparison of the two oppositely facing crystal structures of TtNapA reveals a substantial vertical translation of the conserved four-helix bundle, accompanied by an angular motion of the bundle (a pattern that has been suggested to correspond to the elevator mechanism). Accordingly, in our simulations, we first biased both CVs simultaneously. Indeed, by doing so, we were able to induce conformational changes in TtNapA and reproduced each of the two known states of the antiporter, when starting from the opposite state, with good accuracy. Encouragingly, the free energy landscape associated with the conformational space of the antiporter as a function of both CVs revealed two main free energy basins corresponding to the IF and OF states (Fig. 3).

Notably, both the angular-motion and the vertical-translation CVs were defined with respect to the conserved four-helix bundle in the antiporter’s core domain. This helical bundle has been suggested to contribute the most to the conformational changes underlying the alternating access mechanism in secondary active transporters (5). The simulations based on these definitions managed to reproduce each of the two known states of TtNapA, when starting from the opposite state, lending additional support to the functional importance of the four-helix bundle in CPAs. In further support of its functional importance, the helix bundle is more evolutionarily conserved than the rest of the transporter (*SI Appendix*).

Interestingly, biasing the angular-motion CV, but not the vertical translation, yielded a free energy landscape that was comparable to that obtained by simultaneously biasing both CVs (Fig. 4). Notably, though only the angular CV was biased, vertical translational motions took place as well, demonstrating a coupling between the two modes of motion; this coupling is also manifested in the diagonal shape of the free energy path connecting the IF and OF basins (Figs. 3, 4*A*, and 5*A*). In fact, the accompanying changes in the vertical translation were comparable to those observed in TtNapA’s two oppositely facing crystal structures. Most importantly, the IF–OF conformational shift only occurred when a certain threshold was reached in the angular CV’s dimension, whereas no such dependence on a threshold of motion was observed for the vertical-translation CV (Fig. 4 and *SI Appendix*, Figs. S2 and S3). Specifically, in some of our simulations, the bundle underwent massive vertical translations of up to 18 Å relative to the initial state of the simulation, which is 1.5 times larger than the observed translation in the two oppositely facing crystal structures, yet the transition between states only occurred when the angular-motion CV changed as well (*SI Appendix*, Fig. S3). Evidently, in contrast to the angular-motion CV and despite the extended simulation time (*SI Appendix*, *Material and Methods*), biasing only the vertical-translation CV is not sufficient to induce the relevant conformational changes associated with the IF–OF transition (Fig. 4*B*), resulting in a fragmented free energy surface. The (seemingly) infinitely high barrier separating the IF and OF basins reflects the failure of all but one simulation to alternate between states. This is a natural consequence; an important CV left out would have to be sampled by brute force. Together, these results suggest that, in essence, the angular motion of the core domain’s four-helix bundle drives the shift between the IF and OF conformations and the resulting translation motion.

The free energy landscape obtained from biasing the angular-motion CV alone closely resembles that obtained from biasing both CVs in its overall shape, including the two local minima of the IF basin, as well as the location of the minima and the barrier between them in the angle-translation space (Figs. 3 and 4*A*). According to the free energy landscape, the transition between the IF_1_/IF_2_ and OF states is a result of a 34° to 35° angular motion of the core domain’s four-helix bundle relative to the dimerization domain, accompanied by 8- to 10-Å vertical translation. The latter is comparable to the 11-Å vertical ascent of TtNapA’s binding site—which consists of residues on TM-4 and TM-5 in the core domain’s four-helix bundle—observed when comparing the OF to the IF crystal structures (6). In our simulations, the motion of the entire core domain relative to the dimerization domain showed similar tilting movement to that of the conserved four-helix bundle (33.5°). Relative to the lipid bilayer’s center of mass, the motion of the entire core domain was characterized by a vertical translation of 5.6 to 6.4 Å, while the whole dimerization domain moved vertically only 1.9 to 3.1 Å relative to the membrane. These results are in line with the ∼6-Å vertical ascent of the core domain and the ∼2-Å ascent of the dimerization domain, relative to the membrane, observed when comparing TtNapA’s OF crystal structure to the IF structure (6).

During the transition between the IF and OF states, as the system approaches the free energy barrier separating the two conformations, the antiporter adopts either an occluded or a water-permeable conformation. In the occluded conformation, the ion-binding site, denoted by D157, is inaccessible to water from either the cytoplasm or periplasm. In the permeable conformation, water can access the binding site from both directions. Similar results were recently obtained for the archaeal NhaP antiporter from *Pyrococcus abyssi* (PaNhaP), another member of the CPA superfamily (13). This phenomenon was also observed in MD studies of other transporters and is suggested to occur in all transporters (25).

Notably, the IF crystal structure (PDB entry 5BZ2) differs somewhat from both IF minima predicted by the simulations. This could be explained by the fact that 5BZ2 is a cysteine cross-linking mutant designed to lock the antiporter in an IF conformation. This arbitrary conformation might not necessarily correspond to an energy minimum. Nevertheless, the free energy landscape further indicates that the two equilibrated crystal structures correlate nicely with the IF_2_ and OF free energy minima. In this regard, it is worth noting that, in contrast to procedures like umbrella sampling or targeted MD simulations, where the reaction path is predetermined, in metadynamics, the system is pushed away from its initial state but the reaction path is not set a priori. To conclude, these results indicate that the angular-motion CV alone produces a good approximation of the conformational changes in TtNapA, and possibly other CPAs.

### EcNhaA.

In light of the success of the simulations in reproducing the experimental structural data of TtNapA with reasonable accuracy, we applied the same procedure to the bacterial antiporter EcNhaA. EcNhaA’s structure has been determined only in the protein’s IF state. Biasing the conformational sampling along the angular-motion CV alone allowed the antiporter to alternate between states and enabled us to obtain a model structure for the OF state (Fig. 5). Reconstructing the free energy landscape associated with the conformational space as a function of both the angular-motion and vertical-translation CVs revealed three main basins corresponding to two IF states (IF_1_ and IF_2_) and one OF state of the antiporter (Fig. 5).

Of the two IF conformations, IF_1_ more closely resembles EcNhaA’s crystal structure. Whereas, for TtNapA, the two IF conformations could be considered as a single free energy basin (Figs. 3*A* and 4*A*), for EcNhaA, the two are distinct and structurally different (Fig. 5*A*). The conformational changes observed during EcNhaA’s transition between the IF_1_ and OF states were more moderate than those observed for TtNapA’s transition between these states. Specifically, as EcNhaA alternated between states, the core domain’s four-helix bundle tilted about 19°, and this tilt was accompanied by a vertical translation of roughly 3.2 Å. The entire core domain showed a similar tilting movement of about 17° relative to the dimerization domain. With respect to the membrane’s center of mass, the core domain moved vertically roughly 4 Å, while the dimerization domain moved 1 Å. Similar observations were obtained in a recent computational study of PaNhaP, where a core domain’s tilting movement of about 11° and a translation of 3.5 Å were reported for the transition between the IF and OF states (13). Furthermore, the rearrangements in EcNhaA’s core domain, which manifested mainly as a tilting movement in the plane of the membrane coupled with a relatively small vertical translation (Fig. 5), appear to be consistent with the rocking-bundle mechanism. Similar conclusions were also drawn from a recent hydrogen/deuterium exchange mass spectrometry study for EcNhaA (26).

EcNhaA’s IF_2_ conformation features a 31° tilting movement of the four-helix bundle and a vertical translation of 6.7 Å relative to the OF state. In this regard, it resembles the IF conformations of TtNapA. EcNhaA’s IF_2_ conformation may represent a distinct inactive state of the antiporter, which could be stabilized by regulatory factors such as pH or ligand binding. Alternatively, given that the protein’s binding site is more exposed to the cytoplasm in the IF_2_ conformation, it could represent the antiporter’s IF state. In this case, the IF_1_ conformation as well as the crystal structure would represent intermediate states on the free-energy path leading to the OF state. Finally, comparison of the IF and OF states also revealed two alternative gates that result from movements of the conserved four-helix bundle, which expose the ion-binding site to either the cytoplasm or the periplasm (*SI Appendix*).

The OF conformation proposed by our analysis for EcNhaA differs from a previous model proposed based on symmetry considerations (7) (rmsd 5 Å; *SI Appendix*, Fig. S8), and is in agreement with previous experimental results (*SI Appendix*). To further support this simulation-derived structure, we designed two Cys replacements (G15C_TM-1_ and A142C_TM-4_) in a monomeric Cys-less NhaA variant. We predicted that the two would be close enough to cross-link via a disulfide bond only in the OF state (*SI Appendix*, Fig. S6 *A* and *B*). If cross-linking does occur, it is expected to lock the antiporter in an inactive state under oxidative conditions, as it did (Fig. 7 *A* and *B*). These results could also arise from a nonspecific effect associated with the introduction of cysteine to the protein. The fact that adding reducing agents [βME or dithiothreitol (DTT)] restored activity (Fig. 7 *A* and *B*) confirms that the inactivation resulted from the formation of a disulfide bond, locking the antiporter in an inactive state. This, in turn, supports the simulation-predicted OF conformation. It should be noted that the cross-linking experiments address only the two opposite end-states of the antiporter (IF and OF) but not the intermediates. To address the latter, more profound experimental characterization is required, perhaps using electron paramagnetic resonance spectroscopy.

In light of the structural differences between TtNapA and EcNhaA, it has often been argued that the two transporters might not share the same transport mechanism. Indeed, our results show significant differences in the magnitudes of both the angular and vertical motions between the two transporters, especially in the vertical-translation CV. However, both seem to share roughly the same rocking-bundle transport mechanism.

### Concluding Remarks.

In summary, our results indicate the following: first, the transport mechanisms of TtNapA and EcNhaA have similar features; second, their mechanisms include aspects of both elevator and rocking-bundle mechanisms; and third, the angular motion of the conserved four-helix bundle is sufficient to induce the conformational changes required for the transition between the IF and OF states. Taken together, these results can be interpreted as strongly supporting the rocking-bundle mechanism for ion/proton transport.

## Materials and Methods

### Metadynamics Simulations.

Well-tempered metadynamics simulations (14) were conducted using the GROMACS package (version 5.1.0 and 2019.2) (27) and the PLUMED Library (versions 2.3.0 and 2.5.2) (15). The all-atom CHARMM36-2015 force field for GROMACS (28) was used to describe ions, proteins, and lipids, and the TIP3P model was employed for water molecules (29). Particle-mesh Ewald was used for electrostatics with a cutoff of 1 nm, updating the neighbor list every 10 steps. The temperature was set to 310 K using the Bussi velocity-rescaling thermostat, the pressure kept at 1 bar using a Parrinello–Rahman barostat, and the approximate free NaCl concentration was 100 mM. Two CVs were defined using PLUMED’s ANGLE and DISTANCE commands (15). Gaussians were deposited every 500 time steps for TtNapA and every 250 time steps for EcNhaA. The initial Gaussian height was set to 1.5 kJ/mol and widths to 1.7° (0.03 radians) for the angular-motion CV and 1 Å (0.1 nm) for the vertical translation CV. The bias factor was set to 20 (30). A more detailed description of the systems setup and metadynamics procedure is provided in the *SI Appendix*.

### Cell Resistance, Site-Directed Mutagenesis, and Antiport Activity.

To test cell resistance to Li^+^ and Na^+^, cells transformed with monomeric Cys-less NhaA variant were grown on LBK to A_600_ of 0.5. Samples (2 µL) of serial 10-fold dilutions of the cultures were spotted onto agar plates containing the selective media, modified LB in which NaCl was replaced with the indicated concentrations of NaCl or LiCl at the various pH levels, and incubated for 2 d at 37 °C.

Site-directed mutagenesis was carried out according to a PCR-based protocol (31) with pCL-AXH3 as a template. Everted membrane vesicles were prepared and used to determine the Na^+^/H+ antiport activity as described previously (32, 33). The assay of antiport activity was based upon the measurement of Na^+^/Li^+^-induced changes in ΔpH as measured by acridine orange, a fluorescent probe of ΔpH. The effect of reducing conditions was explored using βΜE or DTT. A more detailed description of the experimental work is provided in the *SI Appendix*.

## Supplementary Material

Supplementary File

Supplementary File

## Data Availability

Original code and structure data created for the study and PDB files for general use have been deposited in GitHub and are available at https://github.com/galmasrati/CPA-MTD-Repository (30).

## Note Added in Proof.

After this manuscript was accepted for publication, the long-sought after structure of a mammalian CPA, NHE9 from horse, came out (34). The manuscript title implies that NHE9 follows an elevator mechanism, which would be different from the results of the two (remotely) homologous bacterial CPAs in this study. However, the elastic network model based on the inward-facing conformation while suggesting a pathway from inward-facing to (putative) outward-facing conformations, does not determine if the transition itself is dominated by rotational or translational degrees of freedom (or both). Further computational as well as experimental studies on both systems might help characterize the transition motion for all proteins in the family.
