# Supplementary material for: An angular motion of a conserved four-helix bundle facilitates alternating access transport in the TtNapA and EcNhaA transporters
Source: Proc Natl Acad Sci U S A. 2020 Nov 30;117(50):31850–60. doi: 10.1073/pnas.2002710117 (PMC7749304; doi:10.1073/pnas.2002710117)
Supplement: Supplementary File [file pnas.2002710117.sapp.pdf]

Supporting Information

An Angular Motion of a Conserved Four-Helix Bundle Facilitates Alternating Access Transport in the TtNapA and EcNhaA Transporters

Gal Masrati[1], Ramakanta Mondal[2], Abraham Rimon[2], Amit Kessel[1], Etana Padan[2], Erik Lindahl[3] and Nir Ben-Tal[1*].

[1] Department of Biochemistry and Molecular Biology, George S. Wise Faculty of Life Sciences, Tel Aviv University, Ramat Aviv 69978, Israel.

[2] Department of Biological Chemistry, The Alexander Silberman Inst. of Life Sciences, The Hebrew University of Jerusalem, Jerusalem 91904, Israel.

[3] Science for Life Laboratory, Stockholm University & KTH Royal Institute of Technology, Sweden.

* Correspondence: Nir Ben-Tal, Web: http://bental.tau.ac.il, Email: bental@tauex.tau.ac.il.

Supplementary Material and Methods

System setup

Computational models derived from the crystal structures of the TtNapA (PDB entries 5BZ2 and 5BZ3) and EcNhaA (PDB entry 4AU5) monomers were inserted into a (simulated) hydrated and equilibrated membrane using gmx membed[1]; overlapping water molecules and lipids were deleted. The membrane consisted of 338 POPE and POPG lipids at a ratio of 4:1 respectively. POPE is the major phospholipid in Gram-negative bacteria such as *T. thermophilus* and *E. coli*, composing 70-80% of the membrane's lipid bilayer, whereas POPG is the second most common lipid at 20-25%[2,3]. Other lipids, such as cardiolipin, usually only account for a maximum of 5% of the membrane. Thus, a lipid bilayer comprising POPE and POPG at a ratio of 4 to 1, respectively, is a good model for the membranes of both

bacteria without making it too complex. TtNapA's PDB entries 5BZ2 and 5BZ3 both represent mutated versions of the antiporter, in which two cysteines were introduced into the wild-type amino acid sequence. Thus, we first rebuilt the mutated positions to reflect the wild-type sequence. Specifically, in 5BZ2 C31 and C130 were replaced with a valine and isoleucine, respectively; and in 5BZ3 C20 was replaced by methionine, while C166 and C326 were both replaced by valine. TtNapA and EcNhaA are both homodimers. Here we used monomers to reduce the computational burden. This choice is justified by the fact that EcNhaA monomers are fully active[4], and that monomers of MjNhaP1—a close structural homologue of TtNapA— have been shown to function independently of each other[5]. Finally, protonation states of titratable residues were determine using PROPKA[6,7] at pH 8. This pH value was chosen, as TtNapA reaches its maximum activity at pH 8[8] and EcNhaA at pH that ranges between 8 and 9[9,10]. Specifically, in EcNhaA no titratable residues were protonated, while in TtNapA Glu74 and Glu265 were protonated. Importantly, the data presented in the main text reflects the deprotonated state of the two aspartic acids in both TtNapA's and EcNhaA's binding site (Asp156-Asp157 and Asp163-Asp164, respectively). The effect of proton binding was explored for TtNapA only, as described below.

Simulations were performed with GROMACS-5.1.0 and GROMACS-2019.2[11], 2 fs time step and bonds involving hydrogens constrained[12]. We applied the CHARMM36-2015 force field for GROMACS[13] to the protein and lipids, and TIP3P model for water molecules[14]. Particle-mesh Ewald (PME) was used for electrostatics with a cutoff of 1 nm, updating the neighbor list every 10 steps. The temperature was set to 310 K using the Bussi velocity-rescaling thermostat, and the pressure kept at 1 bar using a Parinello-Rahman barostat, and the approximate free NaCl concentration to 100 mM. Each system was minimized using steepest descent, followed by unbiased equilibration runs of 155 ns before applying the bias potential as described below.

Metadynamics simulations

Well-tempered metadynamics simulations[15] were employed to study the conformational sampling along two CVs, using the PLUMED-2.3.0 and PLUMED-2.5.2 libraries[16]. These CVs were defined using

the conserved four-helix bundle located at the core domain, consisting of TMs 4, 5, 11 and 12 (Fig. 2). For considerations of symmetry, a parallel four-helix bundle situated at the heart of the dimerization domain was also defined by helices 2, 7, 8 and 9 (Fig. 2). TMs 2, 8 and 9 were selected as they define each antiporter's funnel from the dimerization domain's side; while TM-7, a prominent component of the dimerization interface, was preferred over TM-1, which is more peripheral.

The first CV was an angular motion of the conserved four-helix bundle of the core domain, defined by PLUMED's ANGLE CV[16], which computes the angle between three atoms (Fig. 2a). To that end, three virtual atoms were defined, situated at the periplasmic (a_1) and cytoplasmic (a_2) ends of the core domain's four-helix bundle, and at the cytoplasmic end of a parallel four-helix bundle in the dimerization domain (a_3). Each virtual atom was defined as the center of mass of four C_α atoms situated at the ends of the relevant helices. Specifically, in TtNapA, virtual atom a1 was defined using residues $P115_{TM-4}$, $V167_{TM-5}$, $G342_{TM-11}$ and $E352_{TM-12}$; virtual atom a2 by residues $L135_{TM-4}$, $S146_{TM-5}$, $T323_{TM-11}$ and $L371_{TM-12}$; and virtual atom a3 by residues $L73_{TM-2}$, $A219_{TM-7}$, $G245_{TM-8}$ and $E258_{TM-9}$. In EcNhaA, virtual atom a1 was defined using residues $E124_{TM-4}$, $I171_{TM-5}$, $A346_{TM-11}$ and $L357_{TM-12}$; virtual atom a2 by residues $L141_{TM-4}$, $L152_{TM-5}$, $Q327_{TM-11}$ and $L380_{TM-12}$; and virtual atom a3 by residues $L77_{TM-2}$, $T205_{TM-7}$, $G234_{TM-8}$ and $L251_{TM-9}$. The different angles sampled by the system during the simulation were manifested as an angular motion of the entire core domain relative to the dimerization domain.

The second CV was the vertical translation of the four-helix bundle, computed using PLUMED's DISTANCE CV[16], which calculates the distance between two atoms. Using the COMPONENTS option we biased the distance along the z-axis between two virtual atoms representing the center of mass of the core domain's and dimerization domain's four-helix bundles, b_1 and b_2, respectively (Fig. 2b). In TtNapA, virtual atom b1 was defined using the $\boldsymbol{C_\alpha}$ atoms of residues $T113\text{-}L138_{TM-4}$, $S146\text{-}T173_{TM-5}$, $V318\text{-}A345_{TM-11}$ and $E352\text{-}E379_{TM-12}$; and b2 by residues $E54\text{-}E74_{TM-2}$, $P215\text{-}I233_{TM-7}$, $P237\text{-}L248_{TM-11}$ and $L257\text{-}R279_{TM-12}$. In EcNhaA, b1 was defined by residues $T122\text{-}R147_{TM-4}$, $V148\text{-}Y175_{TM-5}$, $Q326\text{-}V353_{TM-11}$ and $D354\text{-}R383_{TM-12}$; and b2 by residues $L60\text{-}Q85_{TM-2}$, $T205\text{-}K221_{TM-7}$, $S222\text{-}F236_{TM-8}$ and P247-

$A272_{TM\text{-}9}$. Biasing the distance along the z-axis resulted in a vertical translation along the membrane's normal of one domain relative to the other.

The bias potential was applied on either both or only one of the two CVs described above using PLUMED's METAD function[16]. Gaussians were deposited every 500 time steps for TtNapA and every 250 time steps for EcNhaA, in an attempt to reduce simulation time. The initial Gaussian height was set to 1.5 kJ/mol and its width to 1.7° (0.03 radians) for the angular-motion CV and 1 Å (0.1 nm) for the vertical translation CV. These values were equal to less than half the variation of each CV in the last 50 ns of the unbiased equilibration simulations. The bias factor $\gamma\left(\frac{T+\Delta T}{T}\right)$ was set to 20, with the system's temperature being 310 K. The deposited bias was stored on a grid with lower and upper bounds of ±180° for the angular-motion CV, and ±30 Å for the vertical-translation CV. The grid spacing was set to a fifth of the Gaussians' width, that is, ~0.34° (0.006 radians) for the angular-motion CV and 0.2 Å (0.02 nm) for the vertical-translation CV. For TtNapA a total of 19 simulations of 1-to-1.5 μs each were performed. 12 out of these simulations, of 1-to-1.5 $\boldsymbol{\mu s}$ each, were divided into three groups. Each simulation in each group began from either the IF or OF state (Table S1, simulations 1-through-12), and the bias potential was applied to either the angular-motion CV, vertical translation CV or both. 2 additional simulations where only the angular-motion CV was biased were used to assess the effect of additional simulation time on convergence (Table S1, simulations 13-through-14). 4 simulations, of 1 μs each, with bound-protons at TtNapA's binding site (Asp-156 and Asp-157) were utilized to estimate the effect of protein-ion/protons interaction on the resulting FES (Table S1, simulations 15-through-18). And finally, a single 1 μs simulation, where only the vertical translation CV was biased using larger Gaussians, was used to test the bias potential parameters' effect on the results (Table S1, simulation 19). See below for more details.

For EcNhaA four simulations of 0.6 μs each were conducted, where only the angular-motion CV was biased, starting from either the IF or OF states (Table S1). For both antiporters, in all the simulations

that were initiated from a computationally-derived model, the models were simulated in an unbiased simulation for 150 ns, similarly to the crystal structure, before the bias potential was applied. As there is no crystal structure that could be used as reference for the computationally derived NhaA's OF model, we extended its 150-ns relaxation simulation to ensure that the structure maintains an OF conformation, which it did for up to 1 μs. Importantly, on average the antiporter sampled values of both the angular motion and vertical translation CVs within the free energy basin of the OF conformation (Fig. S9).

It is important to note that in metadynamics, the Gaussian function used to employ the bias potential for a given CV is defined by two parameters, its height and its width. The Gaussian's height is set in units of energy for any given CV. However, the Gaussian's width is defined in units that match the CV's units. It follows that for the angular motion CV the Gaussian's width is measured in degrees while for the vertical translation CV it is defined by angstroms. A strict comparison between these two unit scales is not trivial. This might be of importance when choosing the Gaussian width.

The Gaussian's dimensions and particularly its width, determine the resolution of the free energy surface and how fast it is explored. It follows that with sufficient simulation time it is better to choose smaller values to improve the resolution. That being said, if two CVs are examined but in theory one of them ends up having a much stronger bias than the other, there is the risk that effectively the conformational sampling will be almost similar to that obtained in a simulation with only one CV. Weak bias could also explain why, with only one exception, simulations where only the vertical translation CV was biased failed to alternate between states.

Thus, to further confirm that the vertical translation Gaussian's width did not affect the ability of the system to alternate between states, additional simulation was carried out where the Gaussian's width was doubled and set at a value of 2Å. The simulation was initiated from the IF crystal structure, which previously failed to alternate between states, and the vertical translation was biased for 1 μs. As can be seen in Fig. S10, even when the Gaussian's width was doubled, TtNapA failed to alternate between states, consistent with the vertical translation CV being only of secondary importance.

To estimate the free energy as a function of the two CVs, we used PLUMED's HISTOGRAM function[16]. The data collected during the metadynamics simulations were reweighted, with the grid's lower and upper bounds updated based on the actual minimum and maximum values of angles and distances sampled during the simulation. Then the probability density function of each CV was computed[17] and the free energy was estimated using equation (1). Finally, the standard error was estimated from block analysis with four equal blocks, where each simulation was considered as one block.

Individual simulations had been running until at least one transition between states was observed and the free energy barrier's height converged. Two individual simulations, one of TtNapA and one of EcNhaA, where the barrier's height appeared not to converge, were discarded and replaced with new simulations with the same settings. Next, using the data collected from all simulations the convergence of the barrier's height was assessed again using block analysis, where individual simulations were treated as individual blocks (Fig. S11). Finally, to further assess convergence, we carried out two additional simulations of 1 μs each, starting from either the IF or OF crystal structures of TtNapA and biasing the angular-motion CV. Fig. S7, demonstrates that there were no significant changes to the topology of the free energy surface as a result of the extended simulation time, and the correlation coefficient between the two plots is 0.94. The free energy barrier converged to similar value (Fig. S11). Specifically, 16.1 $\pm$ 0.2 kcal/mol for the four-simulation data set, compared to 14.9 $\pm$ 0.2 kcal/mol for the six-simulations data set. We concluded that further simulations are not needed. Simulations in which only the vertical translation CV was biased were extended significantly up to 1.5 $\boldsymbol{\mu s}$ to further assure that any system's inability to alternate between the IF and OF conformations is not merely an artefact due to minor statistical fluctuations. In spite of the much longer simulation time, all but one of these simulations failed to alternate between the two conformations. Moreover, in the one successful attempt, the system hardly sampled the region in conformation space that corresponds to the barrier between the two conformations. This clearly supports the hypothesis that the angular-motion CV is important for the transition; when attempting to

drive the system without controlling this degree of freedom the simulations will in general not find the correct transition state (it would rather require brute-force sampling of a large region of phase space). As a consequence of the close-to-inexistent sampling of the transition, the corresponding free energy barrier separating the IF and OF basins when not driving the angular CV will be so high that transitions do not happen on relevant timescales ("infinitely high").

To further ensure the differences are not due to short-time statistical fluctuations, we estimated the convergence by monitoring the correlation coefficient between the free energy surfaces deduced from consecutive 100 ns intervals, as the simulations progressed. During the last three blocks tested, the correlation coefficient converged to the anticipated value of one, indicating convergence of the estimated free energy surface (Fig. S12). Convergence was also observed for the estimated free energy surface of the simulations where the angular-motion CV was biased (Fig. S12). For these calculations we also attempted to calculate the height of free energy barrier, and showed that the estimated value converged as well (Figure S11). We thus conclude that the simulations converged well.

Finally, to examine the effect of protein-ion/proton interaction on the resulting free energy surface, we carried out four simulations in which the two binding-site aspartates of TtNapA (Asp156-Asp157) were in their protonated state (Fig. S7). Each simulation lasted for 1 μs, starting from either the IF or OF states and biasing the angular-motion CV. The free energy barrier retrieved from these substrate-bound simulations was 11.3 $\pm$ 0.2 kcal/mol. As anticipated, this value is somewhat smaller than the 14.9-16.1 kcal/mol barrier calculated from the four- and six-simulations data set, where Asp156 and Asp157 were in their deprotonated (charged) form. The relatively small energy difference is probably an underestimate of the real change in barrier height between the proton-bound and unbound states, reflecting inherent limitations of metadynamics simulations with predefined CVs. In particular, the CVs chosen in this work (Fig. 2) reflect global changes in the transporter, whereas cation/proton-binding is an extremely local event. To accurately study the energetics of ion binding, more rigorous approaches and different CVs should be used.

Evolutionary conservation analysis

To compute the evolutionary conservation of TtNapA, we exploited a comprehensive phylogenetic analysis of the CPA super family[18]. Specifically, the 500 most divergent sequences in the CPA phylogenetic tree were selected using the phylogenetic diversity analysis tool PDA[19]. These sequences, which represent the immense sequential diversity observed in the CPA superfamily, were aligned using hmmalign[20]. Then, the multiple sequence alignment of residues 6 to 374 was used for ConSurf[21] evolutionary conservation analysis. The N- and C-termini were poorly aligned and thus discarded.

The average conservation score of the four-helix bundle, including loop regions, was computed for residues 112 to 171 and 313 to 374. All other residues were used to compute the average conservation level of the rest of the protein. To compute the statistical significance of the difference in average conservation between the two populations, we carried out a two-sample t-test assuming equal variance with a 95% confidence level.

The analysis revealed that the residues composing the four helix-bundle, including its connecting loops, show an average conservation score of 6.14 according to the ConSurf conservation scale (1 being the most variable and 9 the most conserved)[21]. By comparison, the average conservation score of the remaining residues in the protein is 4.69. This seemingly small difference is highly significant statistically, with a *p-value* of 2.63×10^{-9}.

Plasmids, bacterial strains, and culture conditions

pAXH3[4] is a plasmid expressing His-tagged NhaA. pCL-AXH3 carries Cys-less NhaA. EP432 is an *E. coli* K-12 derivative, which is *mel*BLid, *Δnha*A1::*kan*, *Δnha*B1::*cat*, *Δlac*ZY, *thr*1[22]. TA16 is an *E. Coli* K-12 derivative (*nhaA*+, *nhaB*+, *lac*I^Q) and is otherwise isogenic to EP432[4,22].

Cells were grown either in Luria broth (LB) or in modified LB (LBK) in which NaCl was replaced with KCl. The medium was buffered with 60 mM 1,3-Bis[tris(hydroxymethyl)methylamino]propane (BTP). For plates, 1.5% agar was used. To test cell resistance to Li^+ and Na^+, EP432 cells transformed with the respective plasmids were grown on LBK to A_{600} of 0.5. Samples (2 μl) of serial 10-fold dilutions of the

cultures were spotted onto agar plates containing the selective media: modified LB in which NaCl was replaced with the indicated concentrations of NaCl or LiCl at the various pH levels and incubated for 2 days at 37 °C.

Site-directed mutagenesis

Site-directed mutagenesis was carried out according to a polymerase chain reaction-based protocol[23] with pCL-AXH3 as a template. All plasmids carrying mutations are designated by the name of the mutation.

Isolation of membrane vesicles and assay of Na+/H+ antiport activity with and without reducing conditions.

EP432 cells transformed with the respective plasmids were grown in LBK medium, and everted membrane vesicles were prepared and used to determine the Na^+/H^+ antiport activity as described previously[24,25]. The assay of antiport activity was based upon the measurement of Na^+/Li^+ induced changes in ΔpH as measured by acridine orange, a fluorescent probe of ΔpH. The fluorescence assay was performed with 2.5 ml of reaction mixture containing 50–100 μg of membrane protein, 0.5 μM acridine orange, 150 mM cholineCl, 50 mM BTP, and 5 mM MgCl2, and the pH was titrated with HCl as indicated. When the effect of reducing conditions was explored 10 mM β mercapto-ethanol or 10 mM DTT were added and the reaction mixture was incubated for 20 min at room temperature. Then, the membranes were energized by addition of D-lactate (2mM, pH7 titrated by KOH), quenching of the fluorescence was allowed to achieve a steady state, and then Na^+/Li^+ (10 mM) was added. A reversal of the fluorescence level (dequenching) indicates that protons are exiting the vesicles in antiport with Na^+ regardless of the reducing agent used. As shown previously, the end level of dequenching is a good estimate of antiport-activity, and the concentration of the ion that gives half-maximal dequenching is a good estimate of the apparent Km for Na^+ (or Li^+) of the antiporter[26,27].

Supporting Information

EcNhaA's OF model is consistent with previous experimental results.

A model structure of the OF state of EcNhaA was previously suggested based on symmetry considerations[28]. This model differs significantly (RMSD 5 Å, Fig. S8) from the new OF conformation suggested here. In fact, this previous model appears to be in an occluded state. Comparing the two models, it appears that the simulation-derived OF conformation proposed here is more consistent with previous experimental results. Notably, TM-2 is the only TM that lines both the cytoplasmic as well as the periplasmic funnel. This TM has been thoroughly studied to identify the cation pathway in NhaA[29]. For this purpose, the membrane impermeable SH reagent MTSET {[2(trimethylammonium)ethyl]methanethiosulfonate bromide} was used. MTSET is comparable in size to hydrated Na^+ ion. In intact cells, MTSET can reach NhaA only from the periplasm. Hence, accessibility analyses in intact cells, using cysteine mutagenesis and MTSET have shown that, N64, D65 and M68 on TM-2 are all accessible from the periplasmic funnel; and that F71 and F72, also on TM-2, are situated in a narrow cavity within the antiporter's core[28,29]. These results are fully consistent with the OF conformation that we propose (Fig. S13).

Regarding F71, the symmetry-based OF model of EcNhaA has suggested that this residue is accessible from the periplasmic funnel alone[28]. However, some of the conformation representing EcNhaA's OF free-energy basin suggests that F71 might be exposed in this state, but from the cytoplasmic side (Fig. S13b). Accessibility experiments have further demonstrated that F267 on TM-9 is not accessible to the solvent[30]. In contrast, the symmetry-based OF model suggested that F267 is accessible in the OF conformation[28]. In our simulation-derived structure introduced here, F267 is completely buried (Fig. S13b). Taken together, our results suggest that, compared with existing computational models, the OF conformation proposed here is more consistent with experimental data.

Proposed gating mechanism for EcNhaA

Following the analysis of the amino acids tested in the accessibility experiments and comparing the IF_1 and OF states, residues M68, F71, and F72 seem to form the junction between the antiporter's two discontinuous funnels in both conformations (Fig. S14). These three residues, located on TM-2 in the relatively immobile dimerization domain, interact with a fourth residue in the mobile core domain, where the identity of the latter residue differs between the two extreme conformations. This interaction forms the heart of the gating apparatus. That is, when EcNhaA is in its IF conformation, the hydrophobic interactions between F71, F72 and M68 on TM-2 and M341 on TM-11 form the upper cap of the cytoplasmic funnel (Fig. S14a and c). Conversely, in the OF conformation F71, F72 and M68 interact with I134 on TM-4 to form the base of the periplasmic funnel (Fig. S14b and d). The formation of the two alternative gates results from movements of the conserved four-helix bundle in the core domain. These movements produce two results: (i) they position either M341 or I134 close to the three immobile residues on TM-2, creating a different gate in each case; and (ii) they expose the ion-binding site, also located at the conserved four-helix bundle, to either the cytoplasm or the periplasm. Consistently, all of these amino acids are highly conserved in EcNhaA and its homologues, as shown in a comprehensive phylogenetic analysis of the CPA superfamily[18].

Supplementary Figures and Tables

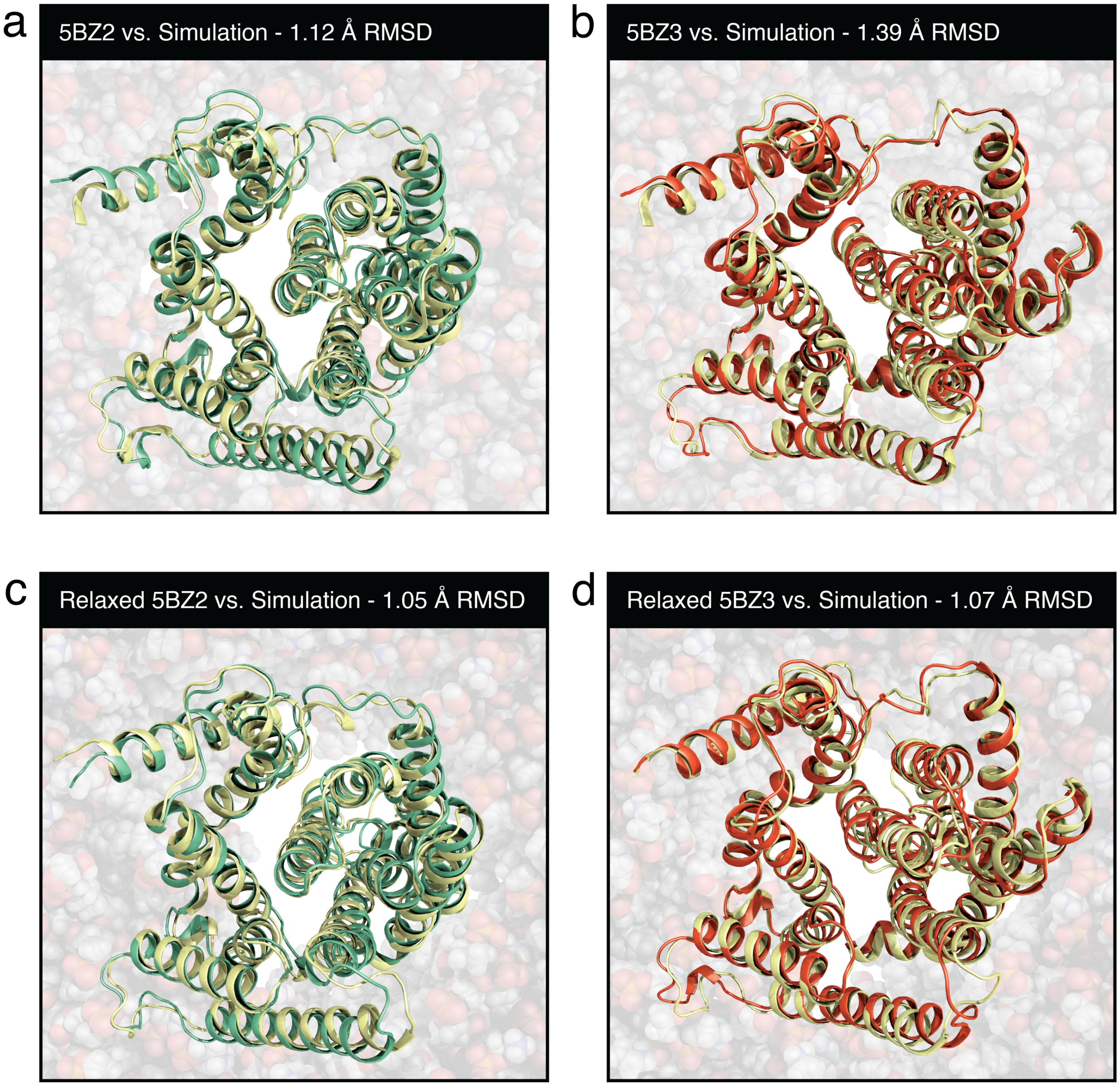

Fig. S1. Superimposition of TtNapA's experimentally determined structures and models derived from the two-CV simulations. (a-b) TtNapA's 5BZ2 (a) and 5BZ3 (b) crystal structures, in cartoon representation and colored yellow, are superimposed onto the closest conformations sampled during the two-CV metadynamics, in green (IF) and orange (OF). The simulations managed to reproduce each of the two known states of the antiporter when starting from the opposite state with a backbone RMSD of 1.12 Å for the IF state and 1.39 Å for the OF state, excluding loop regions. **(c-d)** TtNapA's equilibrated crystal structures superimposed onto the closest conformations sampled during metadynamics, shown as in (a-b). The simulations managed to reproduce each conformation, when starting from the opposite state, with a backbone RMSD of 1.05 Å for the IF state and 1.07 Å for the OF state.

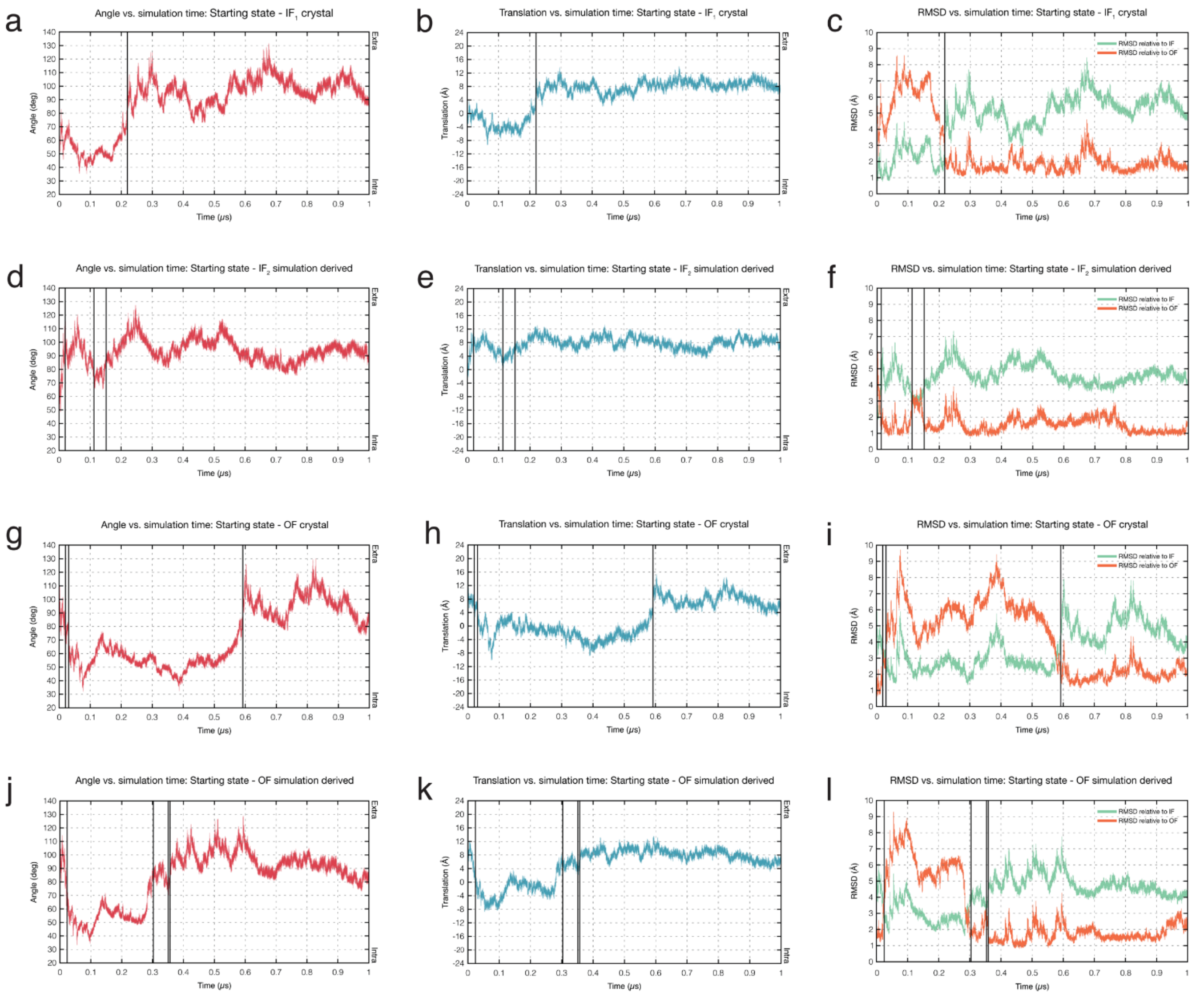

Fig. S2. Biasing the angular-motion CV: angle, vertical translation, and RMSD as a function of simulation time. The left and middle columns show the values of the angle (red) and vertical translation (blue) as a function of simulation time when starting from TtNapA's IF state (**a-b, d-e**) or OF state (**g-h, j-k**), and biasing the angular-motion CV alone. The direction of movement with respect to the extracellular and intracellular sides of the membrane is indicated on the right-top and bottom parts of each plot. The right column depicts the change in backbone RMSD (excluding loop regions) relative to the equilibrated IF state in green and OF state in orange, as a function of simulation time, starting from the IF state (**c, f**) or OF state (**i, l**). In all plots, solid lines denote time-points at which the antiporter assumes a conformation opposite to the simulation's initial state. In plots, depicting RMSD, these lines correspond to time points where the trend in RMSD is reversed. That is, the system alternates between states. As the data show, in all four simulations the antiporter managed to alternate between its two oppositely facing conformations.

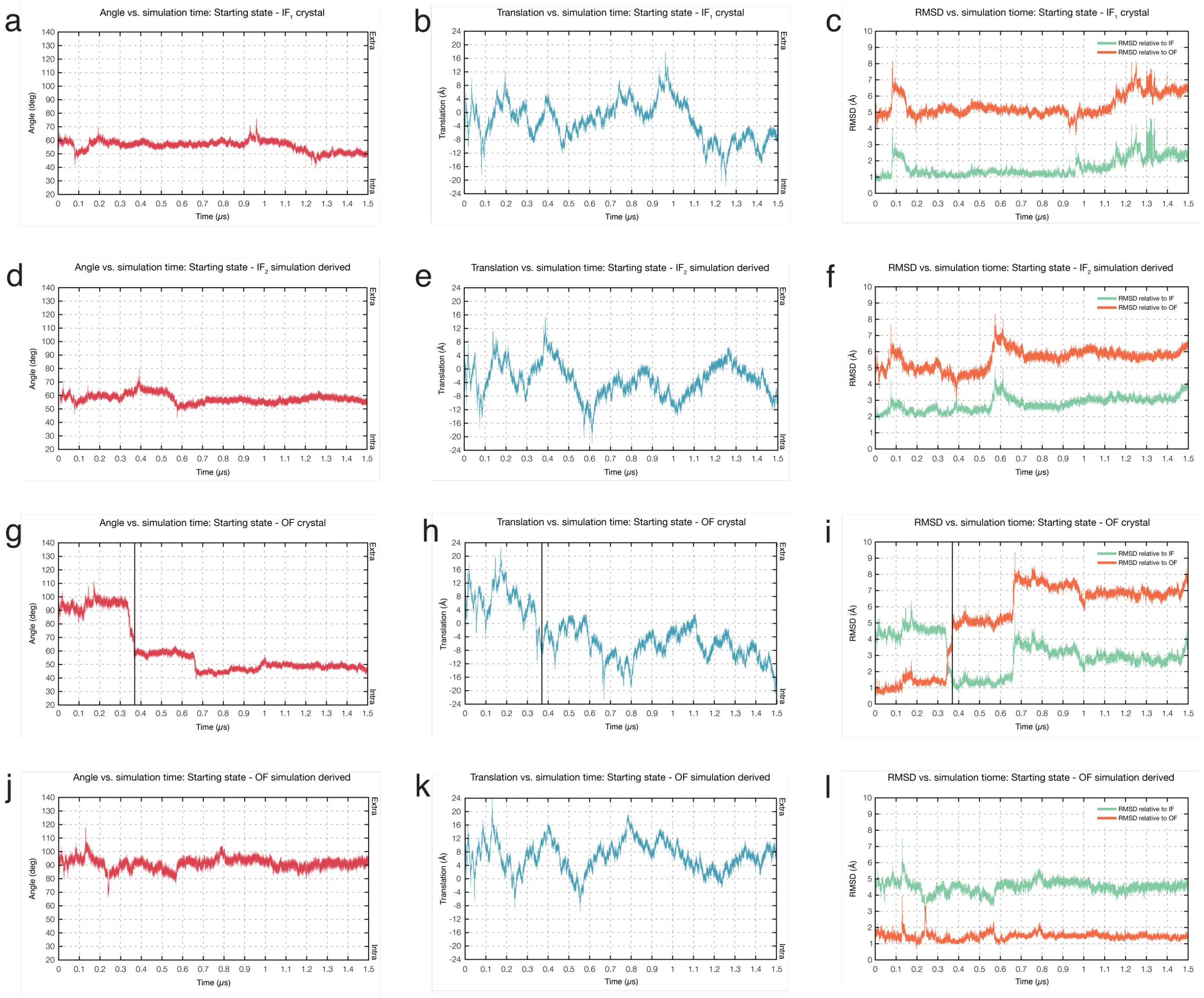

Fig. S3. Biasing the vertical-translation CV: angle, vertical translation, and RMSD as a function of simulation time. The left and middle columns show the values of the angle (red) and translation (blue) as a function of simulation time when starting from TtNapA's IF state (**a-b, d-e**) or OF state (**g-h, j-k**), and biasing the translation CV alone. The right column depicts the change in backbone RMSD (excluding loop regions) relative to the equilibrated IF state in green and OF state in orange as a function of simulation time, starting from the IF state (**c, f**) or OF state (**i, l**). In all plots. The directions of movement and the solid lines are depicted as in Supplementary Figure 2. Notably, in only one out of four simulations, starting from the OF state (**g, h, i**), did the system manage to alternate between conformations. Importantly, despite a translation of up to 18 Å relative to the initial state of the system (for example, plot b at ~0.2 μs), conformational change only occurred when translation was accompanied by an angular motion of the core domain in the relevant direction (**g, h, i**).

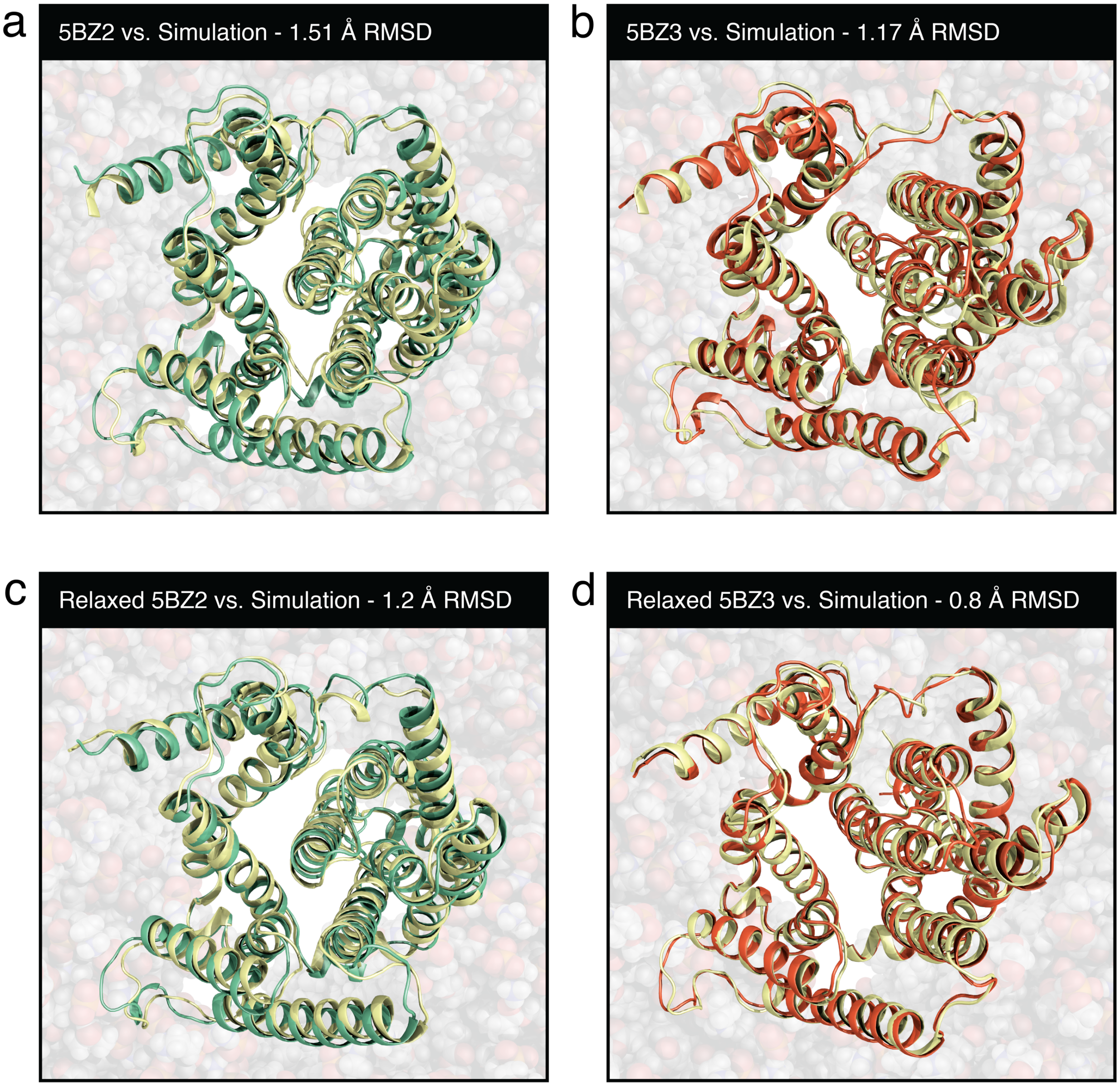

Fig. S4. Superimposition of TtNapA's crystal structures and models derived from simulations where only the angular motion CV was biased. (a-b) TtNapA's 5BZ2 (a) and 5BZ3 (b) crystal structures, in cartoon representation and colored yellow, are superimposed onto the closest conformations sampled during metadynamics, in green (IF) and orange (OF). The simulations managed to reproduce each of the two known states of the antiporter when starting from the opposite state with a backbone RMSD of 1.51 Å for the IF state and 1.17 Å for the OF state, excluding loop regions. **(c-d)** TtNapA's equilibrated crystal structures superimposed onto the closest conformations sampled during metadynamics, shown as in (a-b). The simulations managed to reproduce each conformation, when starting from the opposite state, with a backbone RMSD of 1.2 Å for the IF state and 0.8 Å for the OF state.

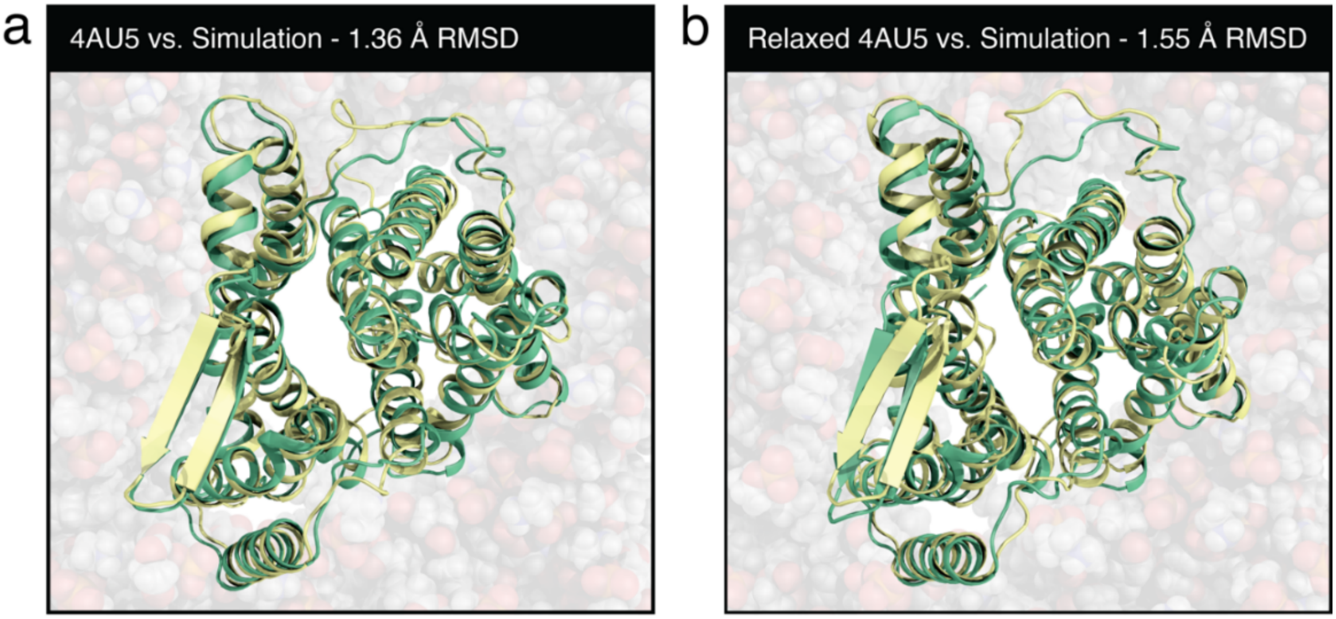

Fig. S5. Superimposition of EcNhaA's crystal structure and the model derived from simulations. (a) EcNhaA's IF crystal structure (4AU5), in cartoon representation and colored yellow, is superimposed onto the closest conformation sampled during metadynamics, in green. The simulations managed to reproduce the IF crystal structure when starting from a simulation-derived OF conformation with a backbone RMSD of 1.36 Å, excluding loop regions. **(b)** EcNhaA's equilibrated crystal structure superimposed onto the closest conformation sampled during metadynamics, shown as in (a). The simulations managed to reproduce the equilibrated structure, when starting from the opposite state, with a backbone RMSD of 1.55 Å, excluding loop regions.

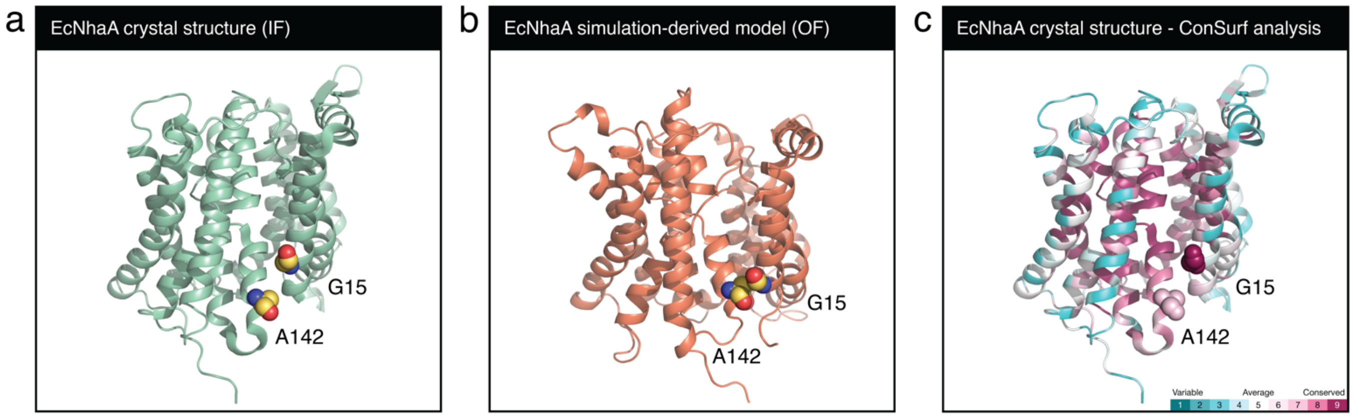

Fig. S6. Designing cysteine replacement mutations in EcNhaA. (a-b) EcNhaA's IF crystal structure (a) and OF simulation-derived model (b) are shown in cartoon representation. G15 and A142, chosen for cysteine replacement, are shown as spheres. The two positions are close enough to form a disulfide bond only in the OF conformation. **(c)** EcNhaA's crystal structure colored according to evolutionary conservation, with cyan-through-maroon representing variable-through-conserved respectively. With conservation grades of 9 and 7 in the 1-through-9 ConSurf scale, both G15 and A142 are evolutionarily conserved.

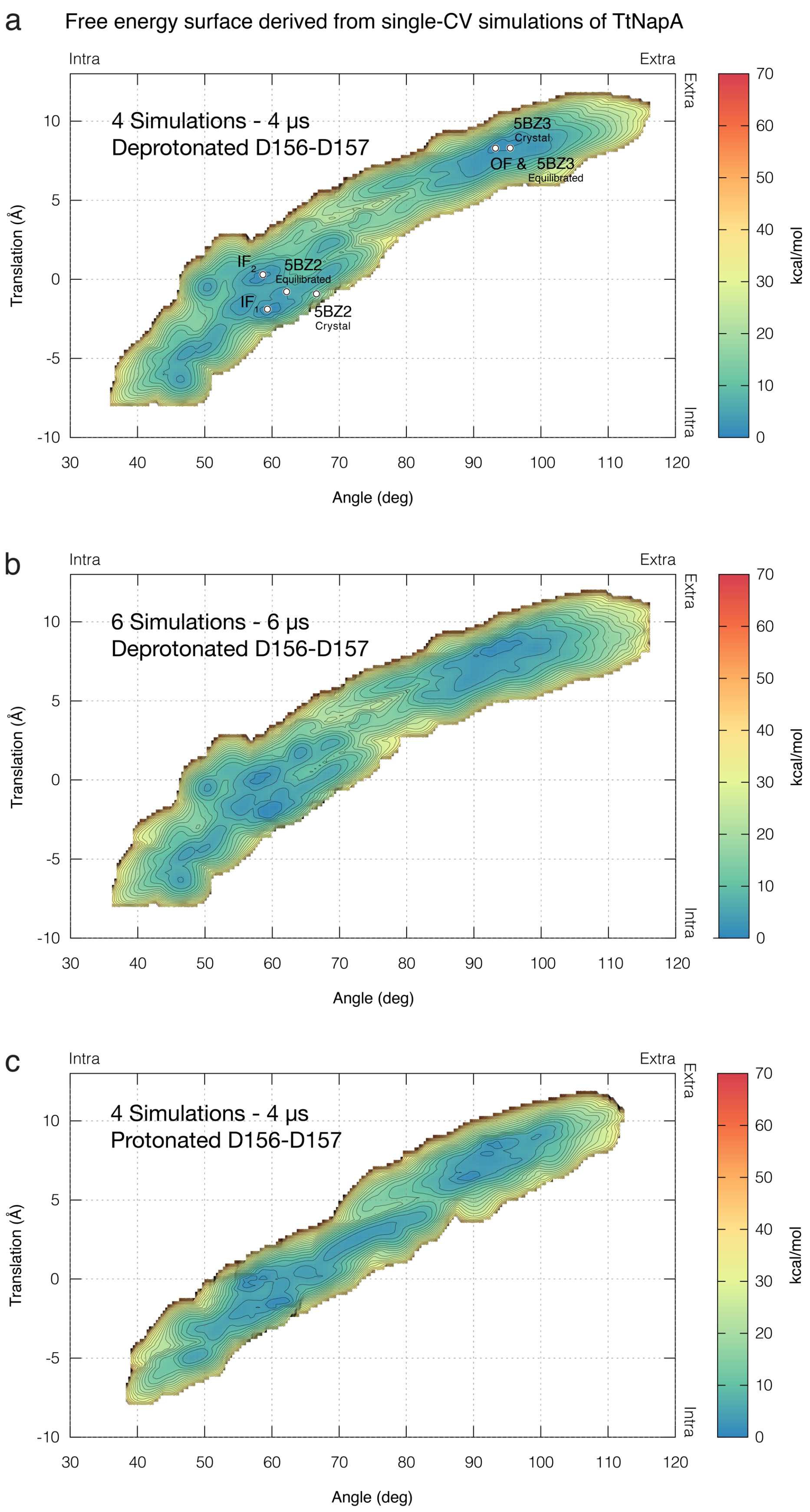
a
Free energy surface derived from single-CV simulations of TtNapA
Intra
Extra
4 Simulations - 4 μs
Deprotonated D156-D157
5BZ3
Crystal
OF & 5BZ3
Equilibrated
IF
2
5BZ2
Equilibrated
IF
1
5BZ2
Crystal
Translation (Å)
Angle (deg)
kcal/mol
b
Intra
Extra
6 Simulations - 6 μs
Deprotonated D156-D157
Translation (Å)
Angle (deg)
kcal/mol
c
Intra
Extra
4 Simulations - 4 μs
Protonated D156-D157
Translation (Å)
Angle (deg)
kcal/mol

Fig. S7. Free energy as a function of vertical translation and angular motion derived from single-CV simulations of 4 and 6-μs with protonated or deprotonated D156-D157. The estimated free energy landscape associated with the conformational sampling of TtNapA as a function of vertical translation (y-axis) and angular motion (x-axis), derived from simulations where only the angular-motion CV was biased. Blue-to-orange color gradient represents low-to-high free energy. Panels **(a)** and **(b)** depict the estimated free energy landscape derived from 4 simulation with total length of 4 μs and 6 simulation with total length of 6 μs, respectively, with deprotonated D156-D157. Panel **(c)** depicts the estimated free energy landscape derived from 4 simulation with total length of 4 μs with protonated D156-D157. Notably, there are no significant changes to the resulting free energy surfaces as a result of the extended simulation time or the protonation state of D156-D157.

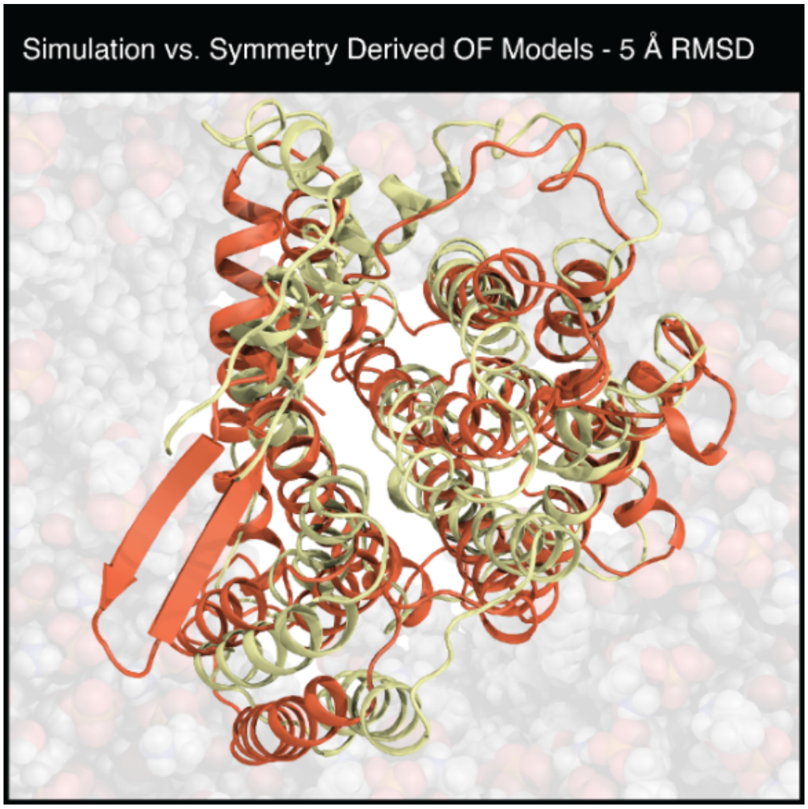

Fig. S8. Superimposition of EcNhaA's simulation- and symmetry-derived OF models. EcNhaA's simulation-derived OF model (orange) and symmetry-derived model (yellow), are superimposed and shown in cartoon representation. The RMSD between the two structures is 5 Å.

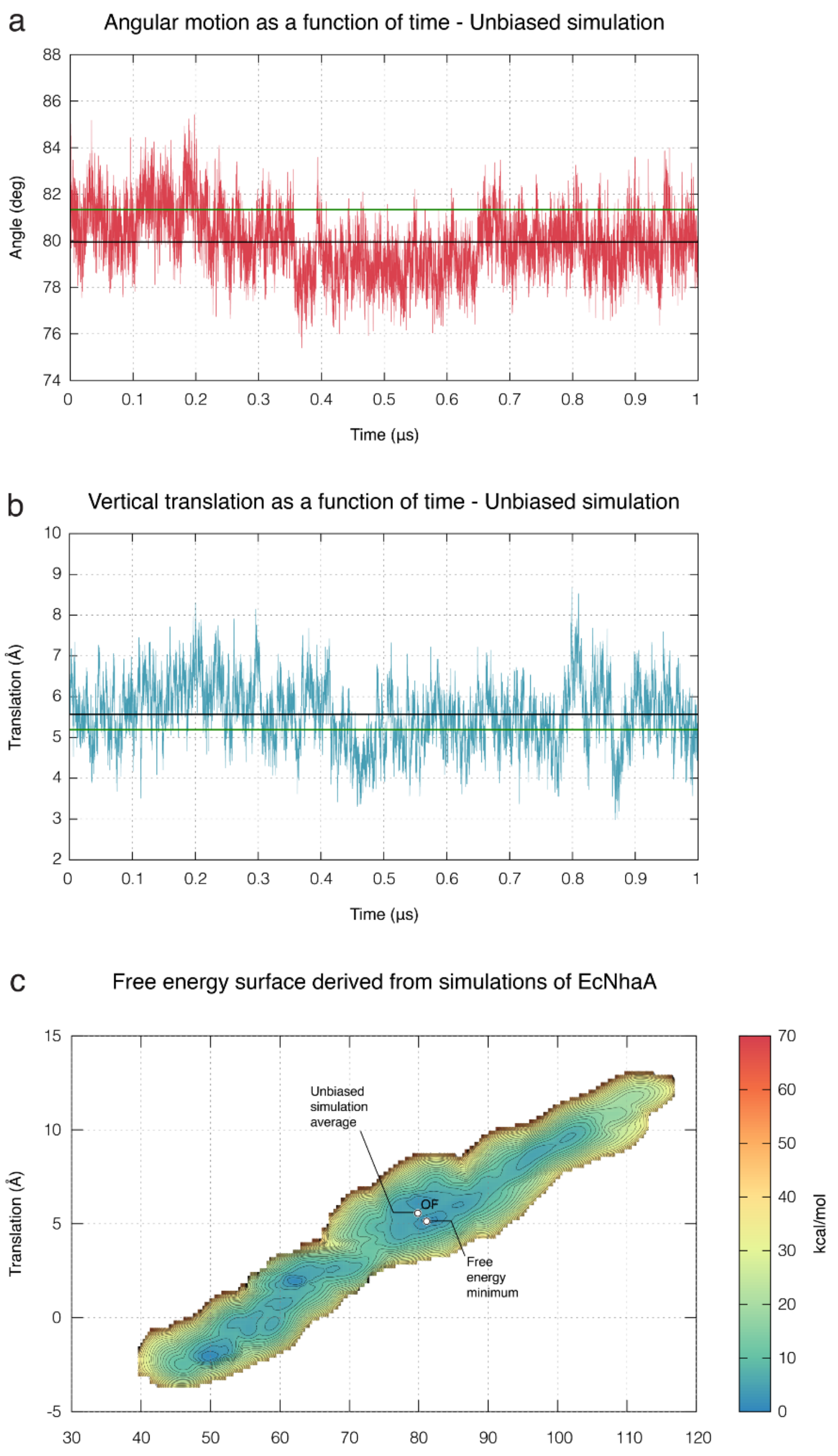
a
Angular motion as a function of time - Unbiased simulation
Angle (deg)
88
86
84
82
80
78
76
74
0
0.1
0.2
0.3
0.4
0.5
0.6
0.7
0.8
0.9
1
Time (µs)
b
Vertical translation as a function of time - Unbiased simulation
Translation (Å)
10
9
8
7
6
5
4
3
2
0
0.1
0.2
0.3
0.4
0.5
0.6
0.7
0.8
0.9
1
Time (µs)
c
Free energy surface derived from simulations of EcNhaA
Translation (Å)
15
10
5
0
-5
30
40
50
60
70
80
90
100
110
120
Unbiased simulation average
OF
Free energy minimum
70
60
50
40
30
20
10
0
kcal/mol

Fig. S9. Unbiased simulation of EcNhaA's simulation-derived OF model. (a-b) The angle (a) and translation (b) values as a function of simulation time when starting from EcNhaA's simulation-derived OF model. The angle and translation values of the free energy minimum calculated from the biased metadynamics simulations are marked with a green line. The average angle and translation values of the unbiased simulation are marked with a black line. **(c)** The estimated free energy landscape associated with the conformational sampling of EcNhaA as a function of vertical translation (y-axis) and angular motion (x-axis). The free energy was derived from single-CV simulations, where only the angular-motion CV was biased. The free energy minimum and the unbiased simulation average values of translation and rotation are marked with circles.

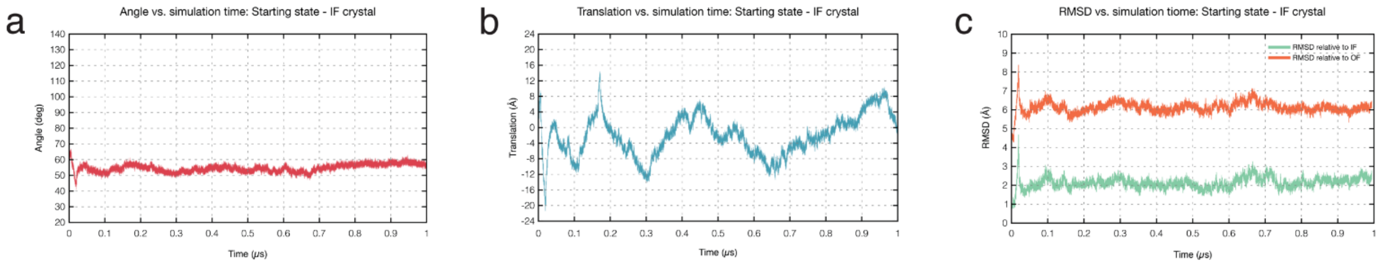

Fig. S10. Biasing the vertical-translation CV with larger Gaussian width: angle, vertical translation, and RMSD as a function of simulation time. (a) and **(b)** show the values of the angle (red) and translation (blue) as a function of simulation time when starting from TtNapA's IF state and biasing the translation CV alone with a Gaussian width of 2 Å. **(c)** The change in backbone RMSD (excluding loop regions) relative to the equilibrated IF state in green and OF state in orange as a function of simulation time. Notably, also with larger Gaussian width the system did not manage to alternate between conformations.

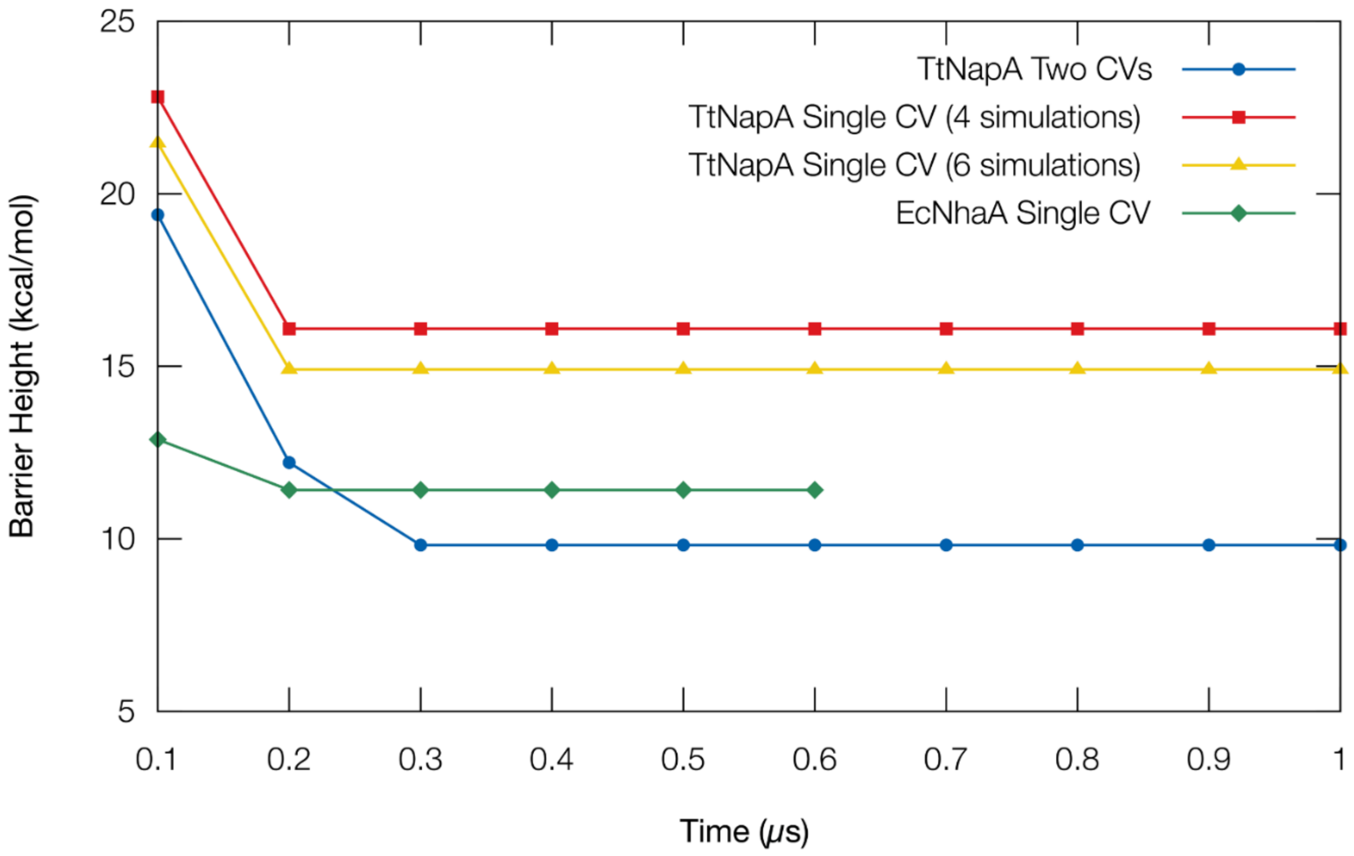

Fig. S11. Minimum barrier height as a function of simulation time. To assess convergence of the metadynamics simulations the minimum barrier height was plotted against the simulation time for double and single (angular motion only) CV simulations of TtNapA and EcNhaA. Note that there is no curve describing TtNapA's simulations in which only the vertical translation CV was biased. The reason is that even after extending TtNapA's simulations up to 1 μs, only in one out of four simulations we observed a conformational shift (Supplementary Figure 3e and f).

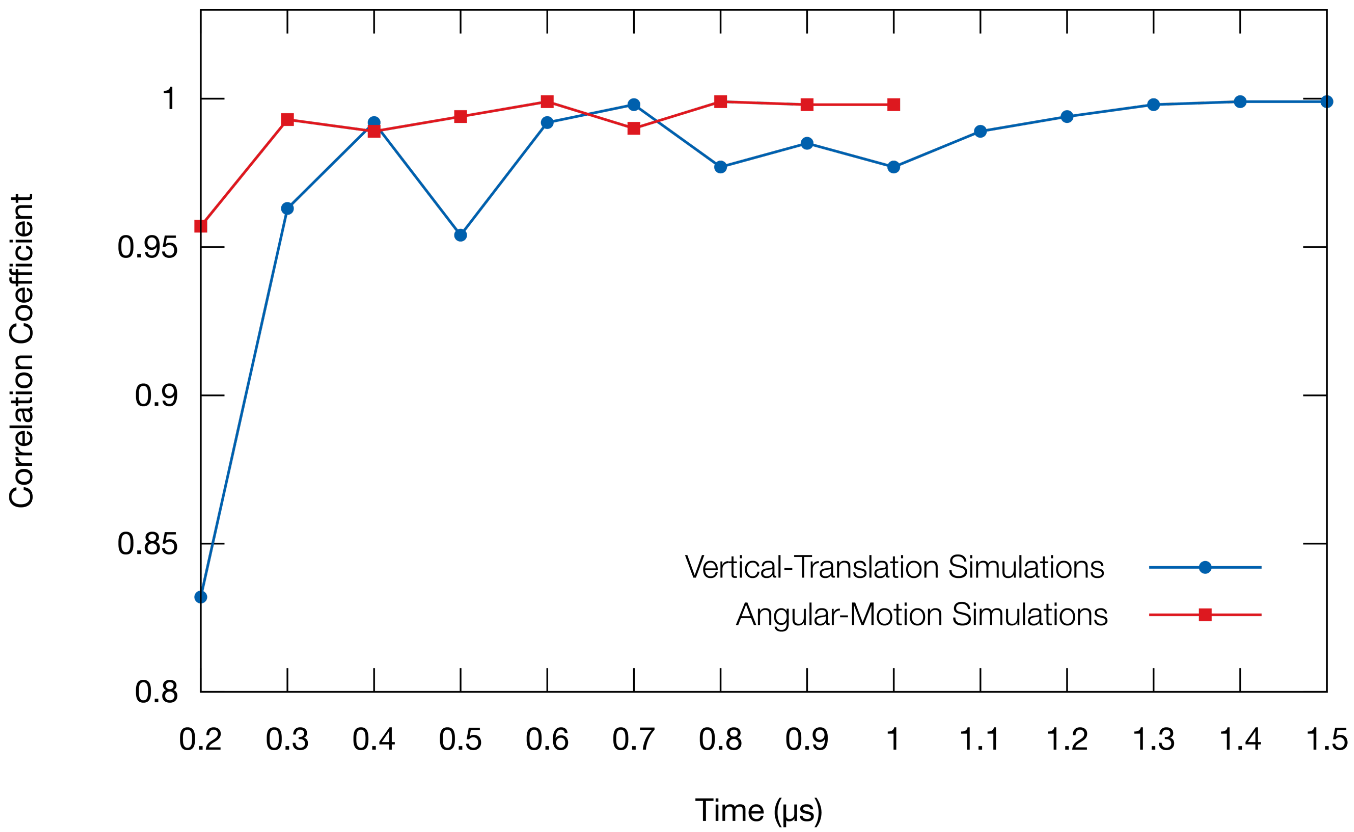

Fig. S12. Correlation between the energy surfaces deduced from consecutive blocks of the simulations, separated by 100 ns intervals, as a function of simulation time. Reassuringly, simulations when biasing the angular-motion CV (red), which dominates the transition between the IF and OF conformations, converge rapidly, resulting in correlation coefficient of 1 already after 0.8 µs. Simulations when biasing the vertical-translation CV (blue), which is secondary in importance, take longer to converge. However, they also saturate to correlation coefficient of 1 (after 1.3 µs).

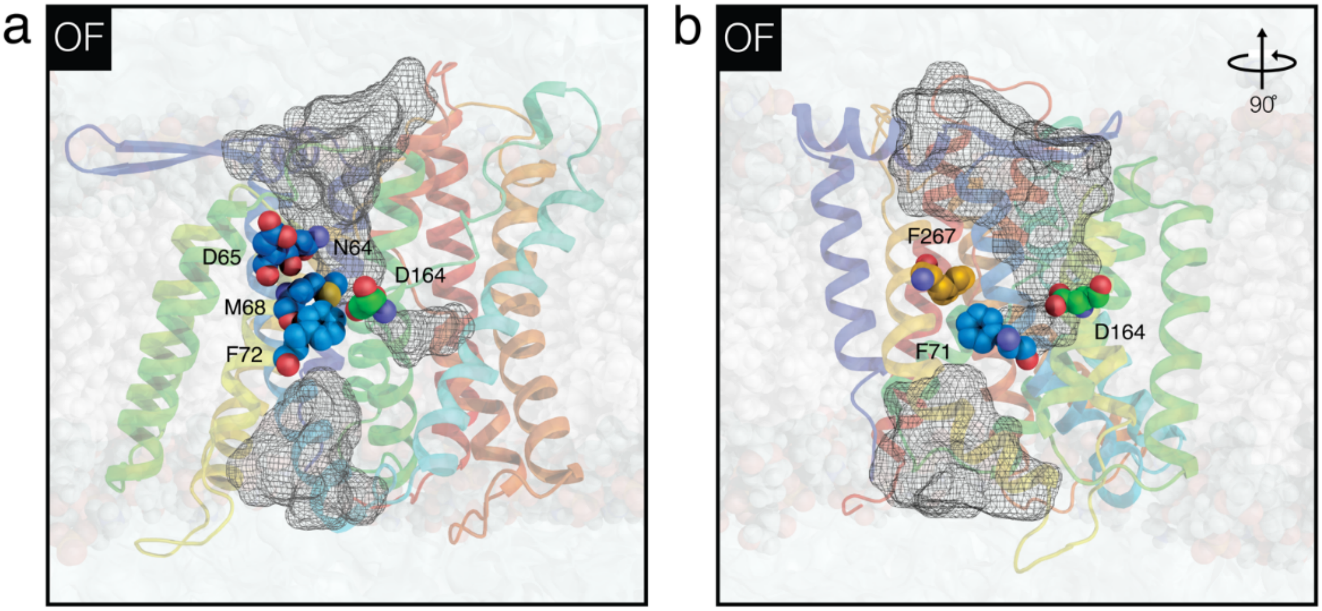

Fig. S13. EcNhaA's OF simulation-derived model is consistent with experiments. EcNhaA in an OF state as seen in the plane of the membrane, with the periplasm at the top and the cytoplasm at the bottom. The two discontinuous funnels leading to the antiporter's binding site, denoted by D164, are shown in mesh representation and colored grey. **(a)** Residues N64, D65, M68 and F72, which line the OF funnel, were shown to be accessible to MTSET[29]. **(b)** F71, also shown to be accessible to *N*-ethylmaleimide, might be exposed from the IF funnel when the antiporter adopts an OF conformation. F267, which, according to experimental data, is not accessible to *N*-ethylmaleimide, is indeed completely buried in the newly proposed OF model[29].

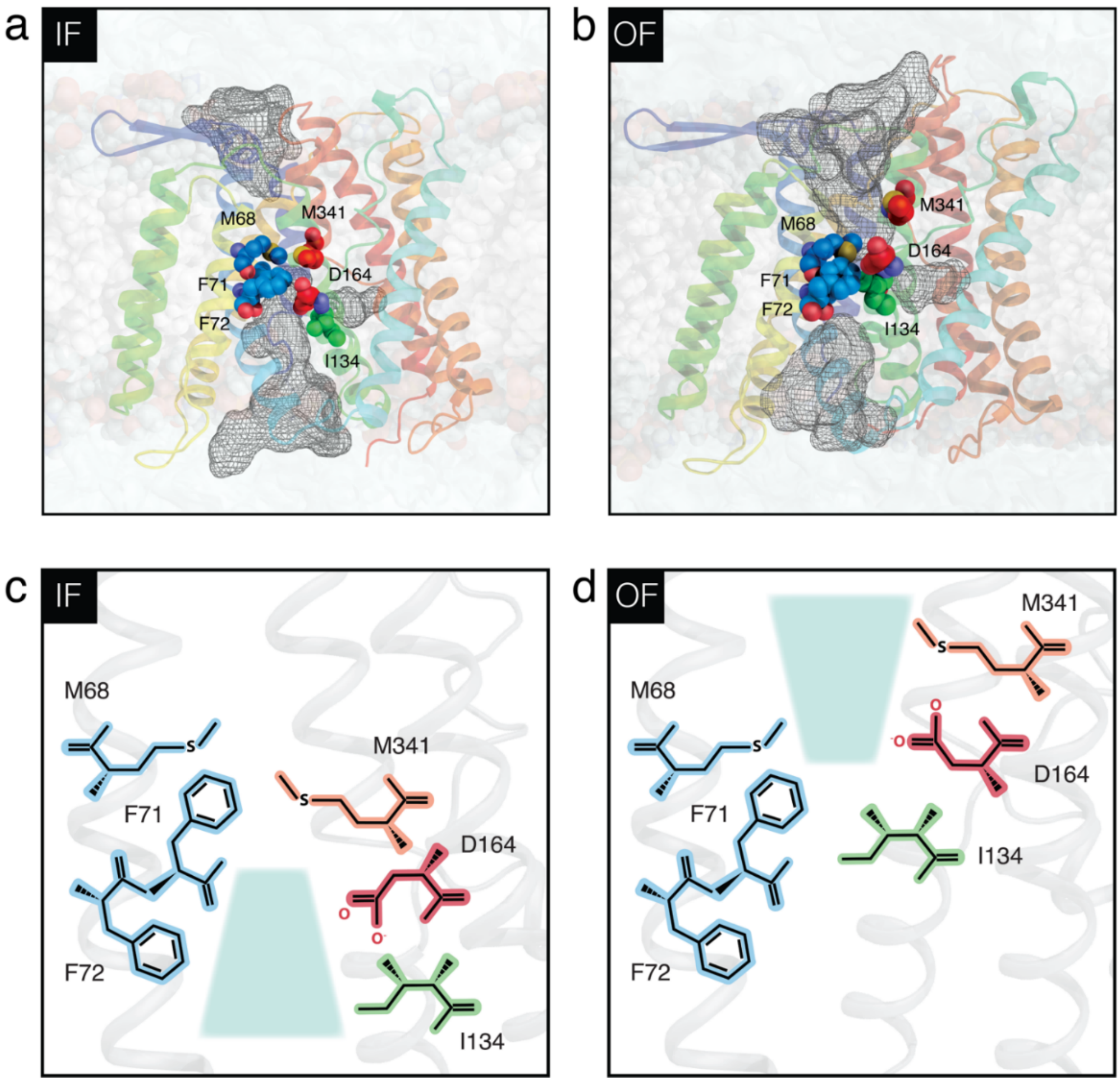

Fig. S14. Proposed gating mechanism for EcNhaA. (a-b) EcNhaA's IF (a) and OF (b) states shown in the plane of the membrane. The two discontinuous funnels leading to the antiporter's binding site, denoted by D164, are shown in mesh representation and colored grey. **(c-d)** Schematic 2-dimensional projections of EcNhaA's core in IF (c) and OF (d) conformations, showing the six residues involved in gating. The IF and OF funnels are represented by cyan trapezoids. As EcNhaA adopts an IF conformation, the hydrophobic interactions between F71, F72 and M68 on TM-2 and M341 on TM-11 form the upper cap of the cytoplasmic funnel, and D164 is accessible from the cytoplasm (a and c). As the antiporter adopts an OF state, F71, F72 and M68 interact with I134 on TM-4 to form the base of the periplasmic funnel, and D164 is accessible from the periplasm (b and d).

Movie S1. Dynamic changes accompanying the IF to OF transition of TtNapA. Simulations-derived step-by-step transition of TtNapA from the IF to the OF state, shown from the periplasm and the membrane's plain. The audio includes detailed description.

ID	Antiporter	Biased CV	Starting State	Conf. Shift	Duration
1	TtNapA	Angular Motion & Vertical Translation	IF_1 - Crystal	Yes	1 μs
2			IF_2 - Simulation derived	Yes	1 μs
3			OF - Crystal	Yes	1 μs
4			OF - Simulation derived	Yes	1 μs
5		Angular Motion	IF_1 - Crystal	Yes	1 μs
6			IF_2 - Simulation derived	Yes	1 μs
7			OF - Crystal	Yes	1 μs
8			OF - Simulation derived	Yes	1 μs
9		Vertical Translation	IF_1 - Crystal	No	1.5 μs
10			IF_2 - Simulation derived	No	1.5 μs
11			OF - Crystal	Yes	1.5 μs
12			OF - Simulation derived	No	1.5 μs
13		Angular Motion Additional Simulations	IF_1 - Crystal	Yes	1 μs
14			OF - Crystal	Yes	1 μs
15		Angular Motion With Bound Protons	IF_1 - Crystal	Yes	1 μs
16			IF_2 - Simulation derived	Yes	1 μs
17			OF - Crystal	Yes	1 μs
18			OF - Simulation derived	Yes	1 μs
19		Vertical Translation With Larger Gaussians	IF_1 - Crystal	No	1 μs
20	EcNhaA	Angular Motion	IF_1 - Crystal	Yes	0.6 μs
21			IF_2 - Simulation derived	Yes	0.6 μs
22			OF - Simulation derived	Yes	0.6 μs
23			OF - Simulation derived	Yes	0.6 μs

Table S1. List of metadynamics simulations. For each simulation performed on either TtNapA or EcNhaA the above table summarizes the CVs that were biased, the starting state of the simulation, whether or not a conformational shift relative to the initial state took place, and the simulation's duration.

References:

1. Wolf, M. G., Hoefling, M., Aponte-Santamaría, C., Grubmüller, H. & Groenhof, G. g_membed: Efficient insertion of a membrane protein into an equilibrated lipid bilayer with minimal perturbation. *Journal of computational chemistry* **31**, 2169–2174 (2010).
2. Dowhan, W. Molecular basis for membrane phospholipid diversity: why are there so many lipids? *Annual review of biochemistry* **66**, 199–232 (1997).
3. Pask-Hughes, R. A. & Shaw, N. Glycolipids from some extreme thermophilic bacteria belonging to the genus Thermus. *Journal of Bacteriology* **149**, 54–58 (1982).
4. Rimon, A., Tzubery, T. & Padan, E. Monomers of the NhaA Na+/H+ antiporter of Escherichia coli are fully functional yet dimers are beneficial under extreme stress conditions at alkaline pH in the presence of Na+ or Li+. *Journal of Biological Chemistry* **282**, 26810–26821 (2007).
5. Vinothkumar, K. R., Smits, S. H. & Kühlbrandt, W. pH-induced structural change in a sodium/proton antiporter from Methanococcus jannaschii. *The EMBO Journal* **24**, 2720–2729 (2005).
6. Søndergaard, C. R., Olsson, M. H., Rostkowski, M. & Jensen, J. H. Improved treatment of ligands and coupling effects in empirical calculation and rationalization of p K a values. *Journal of chemical theory and computation* **7**, 2284–2295 (2011).
7. Olsson, M. H., Søndergaard, C. R., Rostkowski, M. & Jensen, J. H. PROPKA3: consistent treatment of internal and surface residues in empirical p K a predictions. *Journal of chemical theory and computation* **7**, 525–537 (2011).
8. Furrer, E. M., Ronchetti, M. F., Verrey, F. & Pos, K. M. Functional characterization of a NapA Na+/H+ antiporter from Thermus thermophilus. *FEBS letters* **581**, 572–578 (2007).
9. Taglicht, D., Padan, E. & Schuldiner, S. Overproduction and purification of a functional Na+/H+ antiporter coded by nhaA (ant) from Escherichia coli. *Journal of Biological Chemistry* **266**, 11289–11294 (1991).
10. Dwivedi, M., Sukenik, S., Friedler, A. & Padan, E. The Ec-NhaA antiporter switches from antagonistic to synergistic antiport upon a single point mutation. *Scientific reports* **6**, 23339 (2016).
11. Abraham, M. J. *et al.* GROMACS: High performance molecular simulations through multi-level parallelism from laptops to supercomputers. *SoftwareX* **1**, 19–25 (2015).

12. Hess, B., Bekker, H., Berendsen, H. J. & Fraaije, J. G. LINCS: a linear constraint solver for molecular simulations. *Journal of computational chemistry* **18**, 1463–1472 (1997).

13. Bjelkmar, P., Larsson, P., Cuendet, M. A., Hess, B. & Lindahl, E. Implementation of the CHARMM force field in GROMACS: analysis of protein stability effects from correction maps, virtual interaction sites, and water models. *Journal of chemical theory and computation* **6**, 459–466 (2010).

14. Jorgensen, W. L., Chandrasekhar, J., Madura, J. D., Impey, R. W. & Klein, M. L. Comparison of simple potential functions for simulating liquid water. *The Journal of chemical physics* **79**, 926–935 (1983).

15. Barducci, A., Bussi, G. & Parrinello, M. Well-tempered metadynamics: a smoothly converging and tunable free-energy method. *Physical review letters* **100**, 020603 (2008).

16. Tribello, G. A., Bonomi, M., Branduardi, D., Camilloni, C. & Bussi, G. PLUMED 2: New feathers for an old bird. *Computer Physics Communications* **185**, 604–613 (2014).

17. Tiwary, P. & Parrinello, M. A time-independent free energy estimator for metadynamics. *The Journal of Physical Chemistry B* **119**, 736–742 (2015).

18. Masrati, G. *et al.* Broad phylogenetic analysis of cation/proton antiporters reveals transport determinants. *Nature communications* **9**, 1–14 (2018).

19. Chernomor, O. *et al.* Split diversity in constrained conservation prioritization using integer linear programming. *Methods in ecology and evolution* **6**, 83–91 (2015).

20. Eddy, S. R. Profile hidden Markov models. *Bioinformatics (Oxford, England)* **14**, 755–763 (1998).

21. Ashkenazy, H. *et al.* ConSurf 2016: an improved methodology to estimate and visualize evolutionary conservation in macromolecules. *Nucleic acids research* **44**, W344–W350 (2016).

22. Pinner, E., Kotler, Y., Padan, E. & Schuldiner, S. Physiological role of nhaB, a specific Na+/H+ antiporter in Escherichia coli. *Journal of Biological Chemistry* **268**, 1729–1734 (1993).

23. Ho, S. N., Hunt, H. D., Horton, R. M., Pullen, J. K. & Pease, L. R. Site-directed mutagenesis by overlap extension using the polymerase chain reaction. *Gene* **77**, 51–59 (1989).

24. Goldberg, E. B. *et al.* Characterization of a Na+/H+ antiporter gene of Escherichia coli. *Proceedings of the National Academy of Sciences* **84**, 2615–2619 (1987).

25. Ambudkar, S., Mobley, H., Rosen, B. & Zlotnick, G. ION-EXTRUSION SYSTEMS IN Escherichia coli. *Annals of the New York Academy of Sciences* **402**, 455–456 (1982).

26. Schuldiner, S. & Fishkes, H. Sodium-proton antiport in isolated membrane vesicles of Escherichia coli. *Biochemistry* **17**, 706–711 (1978).

27. Tsuboi, Y., Inoue, H., Nakamura, N. & Kanazawa, H. Identification of membrane domains of the Na+/H+ antiporter (NhaA) protein from Helicobacter pylori required for ion transport and pH sensing. *Journal of Biological Chemistry* **278**, 21467–21473 (2003).

28. Schushan, M. *et al.* A model-structure of a periplasm-facing state of the NhaA antiporter suggests the molecular underpinnings of pH-induced conformational changes. *Journal of Biological Chemistry* **287**, 18249–18261 (2012).

29. Herz, K., Rimon, A., Olkhova, E., Kozachkov, L. & Padan, E. Transmembrane segment II of NhaA Na+/H+ antiporter lines the cation passage, and Asp65 is critical for pH activation of the antiporter. *Journal of Biological Chemistry* **285**, 2211–2220 (2010).

30. Tzubery, T., Rimon, A. & Padan, E. Structure-based functional study reveals multiple roles of transmembrane segment IX and loop VIII–IX in NhaA Na+/H+ antiporter of Escherichia coli at physiological pH. *Journal of Biological Chemistry* **283**, 15975–15987 (2008).
